# Pulmonary Rehabilitation with and without a Cognitive Behavioral Intervention for Breathlessness in People Living with Chronic Obstructive Pulmonary Disease: Randomized Controlled Trial

**DOI:** 10.3390/jcm12237286

**Published:** 2023-11-24

**Authors:** Marie T. Williams, Hayley Lewthwaite, Catherine Paquet, Paul Cafarella, Peter Frith

**Affiliations:** 1 Allied Health and Human Performance, Innovation, IMPlementation and Clinical Translation in Health (IIMPACT), University of South Australia, Adelaide, SA 5000, Australia; hayley.lewthwaite@newcastle.edu.au (H.L.); catherine.paquet@fsa.ulaval.ca (C.P.); 2 Centre of Research Excellence Treatable Traits, College of Health, Medicine, and Wellbeing, University of Newcastle, Newcastle, NSW 2308, Australia; 3 Asthma and Breathing Research Program, Hunter Medical Research Institute, Newcastle, NSW 2305, Australia; 4 Faculté des Sciences de l’Administration, Université Laval, Québec, QC G1V 0A6, Canada; 5 Centre Nutrition, Santé et Société (NUTRISS), Institut sur la Nutrition et les Aliments Fonctionnnels (INAF), Université Laval, Québec, QC G1V 0A6, Canada; 6 Centre de Recherche, Centre Hospitalier Universitaire de Québec-Université Laval, Québec, QC G1V 0A6, Canada; 7 Department of Respiratory Sleep Medicine and Ventilation, Southern Adelaide Local Health Network & College of Nursing and Health Sciences, Flinders University of South Australia, Adelaide, SA 5042, Australia; paul.cafarella@sa.gov.au; 8 College of Medicine and Public Health, Flinders University, Adelaide, SA 5042, Australia; p.frith@flinders.edu.au

**Keywords:** cognitive behavior therapy, pulmonary rehabilitation, breathlessness, dyspnea

## Abstract

(1) Background: Most controlled trials of cognitive behavior therapy (CBT) in people living with chronic obstructive pulmonary disease (COPD) have targeted anxiety and depression. (2) Methods: This pragmatic randomized controlled trial explored whether a comprehensive pulmonary rehabilitation program (CPRP) with CBT for breathlessness or social group control (CPRP + SC) significantly improved health outcomes. (3) Results: People with moderate-to-severe COPD were block randomized (CPRP + CBT *n* = 52 or CPRP + SC *n* = 49). Primary outcomes (Hospital Anxiety and Depression scale (HADs), six-minute walk distance (6MWD)) and secondary outcomes (breathlessness, quality of life and habitual physical activity) were assessed before and 1, 6 and 12 months post intervention. Between-group differences were calculated with mixed models for each time point to baseline (intention to treat (ITT)). Participants (*n* = 101, mean ± SD age 70 ± 8.5 years, 54 (53%) males, FEV_1_% pred 47.7 ± 16.3) were similar between groups. Post intervention, primary outcomes did not differ significantly between groups at 1 (6MWD mean difference −7.5 [95% CI −34.3 to 19.4], HADs-A −0.3 [−1.4 to 0.9], HADs-D 0.2 [−0.8 to 1.3]), 6 (6MWD −11.5 [−38.1 to 15.1], HADs-A 1.1 [0.0 to 2.2], HADs-D 0.2 [−0.9 to 1.3]), or 12 months (6MWD −3.8 [−27.2 to 19.6], HADS-A −0.4 [−1.5 to 0.6], HADs-D −0.7 [−1.7 to 0.4]). (4) Conclusions: In this cohort, combining CBT with a CPRP did not provide additional health benefits beyond those achieved by a standard CPRP.

## 1. Introduction

Persistent distressing breathlessness is a common symptom in people with chronic obstructive pulmonary disease (COPD) and impairs participation in physical activity, well-being, and quality of life [1]. This complex symptom results from interactions between multiple systems responsible for breathing regulation and threat recognition [1]. Consequently, persistent breathlessness does not always have a direct relationship with the degree of physical impairment or markers of disease severity [2,3,4]. Even at low intensity levels, physical activity necessitates an increase in ventilation and can cause breathlessness. Where the experience of exertional breathlessness is intensely uncomfortable (and naturally, anxiety evoking [5]) or disproportionate to the intensity of physical exertion, individuals are likely to modify or avoid activity leading to a cycle of habitual inactivity, reduced cardiovascular fitness and earlier breathlessness during physical activity [6]. 

High-level evidence supports pulmonary rehabilitation as a first-line management strategy for people living with persistent breathlessness by improving overall exercise capacity and reducing the anxiety associated with exertional breathlessness [7,8,9]. The proposed mechanisms leading to beneficial health effects of pulmonary rehabilitation in people living with COPD include both physiological (enhanced efficiency of skeletal muscles and respiratory mechanics) [10] and psychological adaptations (reduction in movement-related fear/anxiety, desensitization, and improved tolerance to breathing distress) [5,10].

Psychological approaches that target perceptual processes or anxiety associated with breathlessness have also been recognized as potential therapeutic strategies [5,11,12,13]. In people living with COPD, multiple reviews have been published specifically on the impact of psychological interventions on mental and/or physical health outcomes [11,14,15,16,17,18,19,20,21,22,23,24]. One of the most frequent psychological interventions within these reviews is cognitive behavior therapy (CBT), which is an umbrella term for a range of psychological approaches. These CBT approaches seek to assist people to identify maladaptive beliefs and develop coping skills appropriate to these beliefs and behaviors and may include direct graded exposure inducing a specific symptom [25]. 

To date, most controlled trials of CBT in people living with COPD have targeted generalized anxiety, panic, and depression rather than the sensation of breathlessness [11]. Within these trials, where CBT interventions are combined with exercise training or pulmonary rehabilitation, few studies report consistent and significant between-group benefits for health outcomes [11] or follow-up participants beyond three-to-six months post intervention [22]. 

This pragmatic randomized controlled trial (RCT) sought to determine whether health outcomes were significantly different when a comprehensive pulmonary rehabilitation program (CPRP) included CBT for breathlessness versus a social group control (CPRP + SC). The research hypothesis was that, in people living with COPD, a CPRP including CBT for the sensation of breathlessness would be significantly more effective in improving functional exercise capacity, anxiety, and depression at 1, 6 and 12 months after intervention compared with a CPRP including a social group control.

## 2. Materials and Methods

This pragmatic, block randomized, controlled trial, where assessors were unaware of group assignment, was conducted at a single center in Adelaide, South Australia (National Health and Medical Research Council project grant #1010309). Reporting was informed by guidelines for CONSORT parallel group designs [26] and the extension specific to social and psychological interventions [CONSORT-SPI] [27]. Ethical approval was granted by Human Research Ethics Committees of the University of South Australia (P153/07) and Repatriation General Hospital (P56/07). The trial was registered with the Australian and New Zealand Clinical Trials Registry (ACTRN12611000292976). All participants provided written informed consent. Data components from this trial have been previously published for selected outcomes (baseline [28,29]; baseline and first assessment post intervention) [30]. 

Recruitment for this study was undertaken at the Repatriation General Hospital (RGH), Adelaide, South Australia, between May 2011 and December 2014. All assessments were undertaken in the respiratory function unit of the RGH, while the interventions were delivered in a geographically separate rehabilitation unit. People referred to RGH to undertake the CPRP were eligible for inclusion if they had a clinical diagnosis of COPD, intended to undertake the eight week rehabilitation program and had at least moderate airway obstruction (post-bronchodilator forced expiratory volume in one second (FEV_1_) <80% of predicted and best recorded ratio of FEV_1_ to forced vital capacity < 70% (FEV_1_/FVC < 70% contemporaneous Global initiative for Obstructive Lung Disease (GOLD) statement Grade 2 [31]). Participants were not eligible for inclusion if they had cognitive or memory impairments (Mini-Mental State Examination score < 23/30 [32]), clinically unstable COPD, co-morbidities that were likely to render exercise unsafe, or were registered for lung volume reduction surgery or lung transplantation. All participants completed pulmonary function assessments to confirm the diagnosis and severity of COPD, arterial blood gases and modified Medical Research Council dyspnea scale (mMRC) [33]. 

Prior to the commencement of recruitment, cycles of CPRP planned for RGH (5 to 6 eight-week programs per year, maximum of 24 people per cycle) were prospectively block-randomized to treatment groups using a computer-generated sequence by the original mathematician within the research team (JP). Information specific to each of the treatment groups (timetable and schedule of standard CPRP and additional session) was organized within identical opaque envelopes labelled with the CPRP cycle and dates by one of the investigators (MTW). Neither the mathematician nor the investigator played a role in recruitment, assessments, or provision of any of the interventions. After the provision of written consent and confirmation of eligibility, participants were provided with their group allocation via sealed envelopes, instructed to open the envelope at home, and asked not to disclose their allocation to staff involved in assessments. All assessments were undertaken by study staff who were unaware of group allocation and who played no part in the provision of either intervention. As part of the standard practice for people undertaking CPRP, pharmacological management was reviewed and adjusted as required by a respiratory physician.

### 2.1. Interventions

Participants in both groups received the same center-based CPRP. The eight-week program adhered to the recommendations of the contemporaneous Australian guideline for COPD management specific to pulmonary rehabilitation. The CPRP included twice weekly, 45 min, outpatient, group-based exercise sessions supervised by a physiotherapist (minimum 30 min of combined aerobic and resistance circuit training) and two one-hour self-management education sessions each week (lecture-based). Prescription of exercise intensity was derived from pre-CPRP six-minute walk tests (6MWT; treadmill speed 80% 6MWT walking speed, stationary cycling work rate (Watts) 60% of the peak work rate estimated from 6MWT distance achieved) or repetition maximum (60–80% RM). Exercise intensity was titrated and monitored to achieve a 3 to 4 on the modified BORG 0–10 scale (“moderate to somewhat severe”). Participants were encouraged to exercise at home (walking program) for at least one further session each week. Self-management education sessions were provided by health professionals employed by the RGH (physiotherapist, psychologist, occupational therapist, respiratory nurse, and dietician). Upon completion of the 8 week CPRP, all participants were advised and encouraged to continue exercising (local gyms, fitness centers, and walking programs). The standard CPRP was not altered to accommodate this trial. Participants in both groups attended an additional one hour session (CBT or SC) each week.

### 2.2. Cognitive Behavioral Therapy Group (CPRP + CBT)

The CBT program (BREVE: Breathing: Recognize sensations, Explore thoughts and beliefs, Validate whether thoughts are useful or harmful, Evolve and change behavior) has been previously reported [34]. Briefly, the CBT program was designed to run parallel to the standard eight-week CPRP as a series of eight modules supported by a workbook. Each module included an education component, individual reflective activity, practice tasks for the supervised exercise sessions and homework activities involving the practice of cognitive strategies during activities associated with breathlessness. A psychologist, qualified and experienced in CBT for the management of chronic pain who had not previously been employed by the trial site and was naive to both the BREVE program and outcome measures, was employed for this study. Each week of the CPRP + CBT intervention, the psychologist facilitated a one hour, group-based session, attended the physiotherapist-supervised exercise sessions to facilitate participants’ individual practice of cognitive tasks while breathless and confirmed/reviewed specific individual weekly goals and homework practice tasks. The CBT program did not replicate information included within the standard self-management education sessions for CPRP. Participants were provided with the opportunity to give anonymous written feedback specific to the BREVE sessions on completion of the final week of the program (Supplementary Materials).

### 2.3. Social Group (CPRP + SC)

Participants randomized to the standard CPRP attended a one-hour social group (SC) session each week to match the time spent by participants in the active intervention. This group session was facilitated by a person living with COPD who had previous experience as a consumer representative and no prior experience of CBT. Prior to trial commencement, this facilitator was orientated and briefed about the session intent by members of the research team (MTW, PC). In this group session, participants were provided with light refreshments and were encouraged to socialize and discuss general events. No additional formal health or lifestyle education was provided during this session.

### 2.4. Outcomes

Participants were evaluated within the month prior to commencing CPRP (baseline) and 1, 6, and 12 months after intervention. While the CBT intervention used in this RCT targeted the perception and cognitions associated with breathlessness, altering the experience of breathlessness without subsequent improvements in functional exercise capacity or anxiety/distress was unlikely to reduce health service usage. The predefined primary outcome measures were changes in the distance achieved during the 6MWT (minimum important difference (MID) 30 m, 95% confidence intervals (95% CI) 25 to 33 m [35]) and the Hospital Anxiety and Depression scale [36] (HADs-A MID −1.6 points, range −2.0 to −1.1; HADs-D MID −1.6 range −1.8 to −1.5 [37]). Two standardized 6MWTs, including pre–post Borg scale for Rating of Perceived Exertion (0 to 10), were undertaken with maximum distance achieved used for analysis [38]. HADs subscale scores (range 0 to 21, higher scores denoting greater likelihood of clinically important anxiety or depression) and case thresholds (score 0 to 7 no probable case, 8 to 10 probable case, ≥11 case) [36] were used in analyses.

Secondary outcomes reflected several health domains. At the time of planning this pragmatic RCT, two multidimensional instruments for breathlessness assessment had become available: Dyspnea-12 (published 2010) [39] and a pre-publication version of the Multidimensional Dyspnea Profile [40], though MIDs had not yet been estimated. Scoring for both instruments was as per developers’ recommendations (higher scores reflect greater intensity or distress) with findings interpreted using MIDs specific to people living with COPD as recommended by Ekstrom et al. (2020) [41]. The Self-administered Chronic Respiratory Disease Questionnaire (CRQ, higher scores reflect better health-related quality of life [42], MID 0.5 [43]) assessed the impact of breathlessness on respiratory-related quality of life. 

Habitual activity was assessed using accelerometry (Actigraph GT3X+ accelerometer (GT3X+) Actigraph, Pensacola, FL, USA) and self-reported use of time (Multimedia Activity Recall for Adults and Children (MARCA)) [44] to reflect time spent and nature of sedentary and physical activity. Accelerometers were worn for 24 h (except for water-based activities or if intolerable during sleep) for seven days following study assessment appointments, with participants maintaining a log of non-wear and sleep periods (details concerning protocol and data management have been previously reported [44]). On two occasions during the seven-day monitoring period, participants completed an interviewer-led MARCA interview to recall time use over four full days, one of which was required to be a weekend day [44,45]. While the MARCA allows estimates of daily physical activity level, our interest in this study was average time per day (minutes) across nine mutually exclusive domains (Physical Activity, Screen time, Transport, Quiet time, Self-care, Sociocultural, Work/Study, Chores, Sleep) [44]. 

All participants were invited to, and instructed in, how to complete a symptom diary [46] between post-intervention assessments at 1 and 12 months. An average symptom severity score was calculated based on the number of days symptoms changed and health care decisions [Supplementary Materials]. Co-morbidities on enrolment to the trial (COPD-specific co-morbidity test (COTE) index score) [47] and healthcare usage (emergency department attendances and RGH hospital admissions) between date of baseline and final 12-month assessments were collated from medical record review. After completion of the final 12 months assessment, an individualized summary report (changes in and interpretation of 6MWD, CRQ-D, HADs-A/D outcomes over time reviewed by the respiratory physician (PF)) was provided to participants with a follow-up exit interview scheduled (phone-based, interviewer (MTW) assisted questionnaire) to clarify the information provided in the summary report and to seek feedback on experience of CPRP and CBT or social group sessions. 

### 2.5. Sample Size Estimate

Both intervention groups were expected to improve as a result of participation in the CPRP and our hypothesis focused on the magnitude of change over time (baseline to each of the three post-intervention assessments between groups). A priori sample estimates were based on changes in primary outcomes for the distance achieved in the 6MWT and HADs scores. Using mixed-effects models (Supermix, Version 1.1 2008, Scientific Software International, Lincolnwood, IL, USA) for a small- to medium-effect size (0.15), the sample sized estimate (alpha = 0.05, beta = 0.20) was 62 (31 per group at each of the four assessment points) with a recruitment target of 120.

### 2.6. Data Management and Analysis

Statistical analysis was performed using SAS, Version 9.4 (SAS Institute, Cary, NC, USA). Intention-to-treat (ITT all eligible enrolled participants) and per protocol (PP participants that attended at least one scheduled CPRP session) analyses were undertaken. Differences between groups (primary and secondary outcomes) for change over the trial period were assessed by linear mixed models (mixed-model growth curve analyses using maximum likelihood estimation, SAS Proc Mixed) [48]. Growth curves were used to model data available for each participant, allowing participants who discontinued the study or missed assessments to be included in the longitudinal analyses without imputation of data for the missing observations [49,50,51]. Models were created for both primary and secondary outcomes (significance *p* ≤ 0.05) which included treatment group, time, group x time interaction adjusted for baseline values of the outcome with a random intercept across participants and an autoregressive correlation matrix. Covariates within the fully adjusted models included pre-intervention values for sex, age, body mass index, COTE score ≥ 4, probable case of clinical anxiety or depression (HADs score ≥ 8), FEV_1_% predicted, smoking status pre-CPRP and total number of intervention sessions attended. Generalized linear mixed models were used for positively skewed outcomes using the Poisson distribution (MVPA) and with relative risk calculated. Logistic regression was used when a high percentage of zero values led to non-convergence of the models. For these models, outcome variables were dichotomized (0, ≥1) and ODDS ratios calculated.

### 2.7. Changes to Trial Outcomes after Protocol Pre-Registered

Three changes occurred after the original protocol was registered with ACTRN. (1) In 2015, the clinical site added a symptom-limited cardiopulmonary exercise test as a basis for exercise prescription, necessitating cessation of recruitment before the target sample size was achieved; (2) the untimely death of the original mathematician within our research team required the recruitment of a different statistician; (3) per protocol analysis was undertaken in addition to the planned ITT approach.

## 3. Results

**Figure 1:**
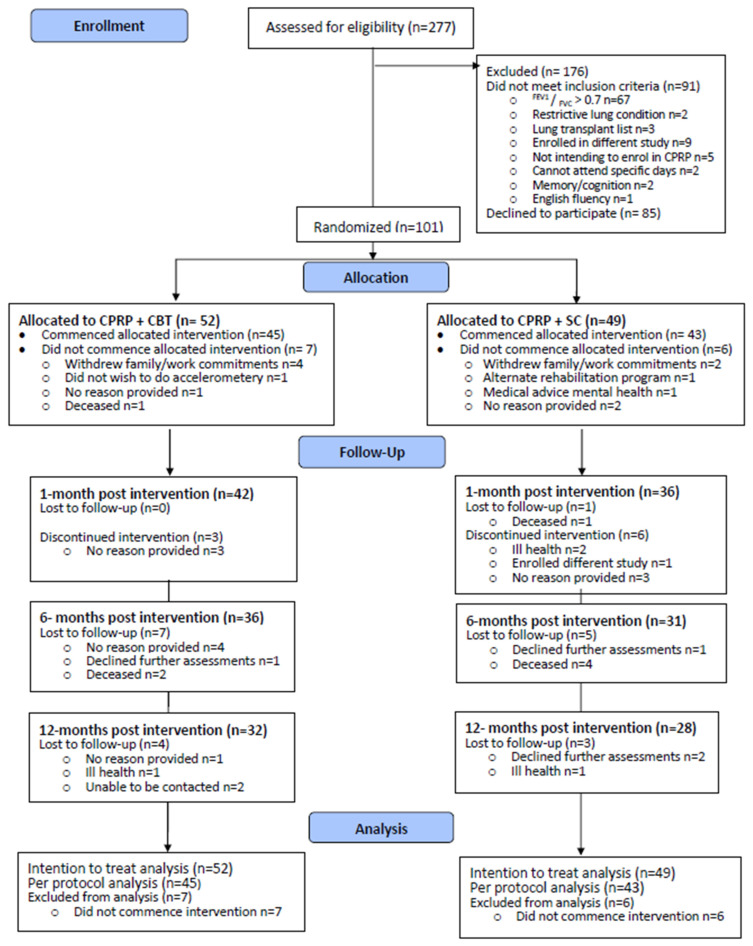
Flow of participants through the trial. CPRP—comprehensive pulmonary rehabilitation program, CBT—cognitive behavior therapy, SC—social group control.

Of the 277 persons screened to participate in the trial, 106 provided written consent, with 101 participants meeting eligibility, enrolled, and randomized (Figure 1, overall uptake rate = 36%; uptake rate of those eligible = 60%). Of the 85 persons declining participation, 66 met GOLD Grade 2 or greater severity. There were no statistically or clinically important differences between individuals who were eligible for participation but declined (*n* = 66) and those who participated in the trial (*n* = 101) [28]. At baseline, primary outcomes displayed little variability between CPRP cycles (intraclass correlation coefficients between 0 and 0.05 for grouping effects of the block randomization). Attrition rates increased across the 12-month trial (end trial attrition CPRP + CBT 38.5%; CPRP + SC 42.8%). Accordingly, primary outcome data availability decreased; 1 month post intervention (CPRP + CBT HADs 77%, 6MWD 65%; CPRP + SC HADs 61%, 6MWD 63%) and 12-month post intervention (CPRP + CBT HADs 54%, 6MWD 48%; CPRP + SC HADs 57%, 6MWD 45% Supplementary Materials). 

Table 1 summarizes the characteristics of participants allocated to the two intervention arms on entry to the study. Groups were comparable, except for the number of participants within mMRC grades (χ^2^ (1, *n* = 101) =13.81, *p* = 0.01) and meeting threshold criteria (borderline or case) for HADs-Depression (CPRP + CBT *n* = 9, 17.3%; CPRP+ SC *n* = 20, 40.8%, χ^2^ = 6.58, *p* = 0.04). Very few participants accrued activity counts that indicated vigorous activity during accelerometry wear days (CPRP + CBT *n* = 2, CPRP + SC *n* = 1), all averaging less than one minute per day. Less than half of the participants entering the study submitted completed symptom diaries for the eight months post intervention (CPRP + CBT *n* = 25, 48%, CPRP + SC *n* = 19, 39%), with no difference between average symptom severity scores (Supplementary Materials). Across this trial, the frequency of emergency department presentations (CPRP + CBT *n* = 21, 40.1%, CPRP + SC *n* = 25, 50.0%), hospital admissions and number of participants admitted to hospital for any cause (CPRP + CBT *n* = 20, 38.5%, CBPR + SC *n* = 22, 44.8%) were comparable (Supplementary Materials).

### 3.1. Primary Outcomes

**Figure 2:**
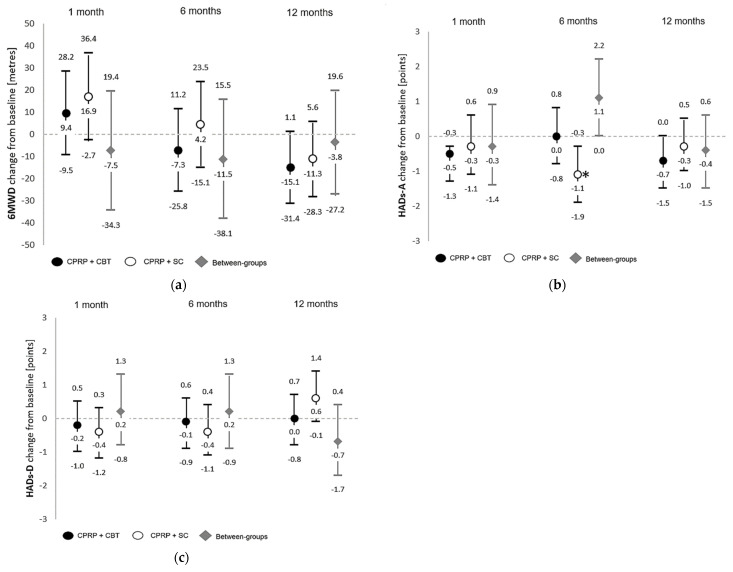
Within- and between-group differences (mean and 95% confidence intervals) from baseline at 1, 6, and 12 months post intervention for primary outcomes where (**a**) presents changes in 6 minute walk distance (meters), (**b**) presents Hospital Anxiety and Depression scores (Anxiety) and, (**c**) presents Hospital Anxiety and Depression scores (Depression). Analysis reflects intention to treat, with fully adjusted models. CPRP—comprehensive pulmonary rehabilitation program, CBT—cognitive behavior therapy, SC—social group control, * significant *p* ≤ 0.05.

Figure 2 summarizes the results of the ITT analysis of between- and within-group changes in distance achieved in the 6MWT and HADs. Per-protocol analyses were consistent with ITT analysis (ITT and PP data presented in Supplementary Materials). There were no statistically significant or clinically important (MID) differences between intervention arms at follow-up assessments for primary outcomes. 

Within both groups, there were very modest increases in distance achieved in the 6MWT at 1 month post intervention, which were not maintained at 6 or 12 months. Within the CPRP + SC group, there was a significant improvement in HADs-A at six months post intervention (Figure 2b; mean −1.1 ± 0.4 *p* = 0.01 CPRP + SC), which fell just within the range for clinically important (MID) improvements. There were negligible changes in mean HADs-D scores for both groups at each assessment post intervention.

### 3.2. Secondary Outcomes

**Figure 3:**
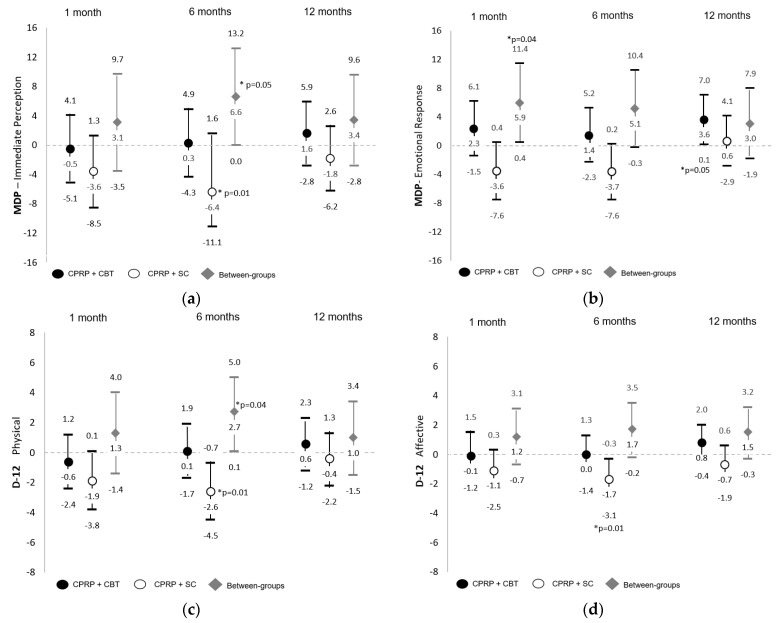
Subdomain scores for multidimensional breathlessness outcomes. Within- and between-group differences (mean and 95% confidence intervals) from baseline at 1, 6 and 12 months post intervention where: (**a**) Multidimensional Dyspnea Profile (MDP)—Immediate Perception; (**b**) MDP—Emotional Response; (**c**) Dyspnea-12 (D-12) Physical; (**d**) D-12 Affective. CPRP—Comprehensive pulmonary rehabilitation program, CBT—cognitive behavior therapy, SC—social group control, * significant *p* ≤ 0.05.

Multidimensional breathlessness outcomes. Results of the ITT and PP analyses of between- and within-group changes for MDP and D-12 scores are detailed in Supplementary Materials. Figure 3 presents subdomain score results for both instruments (ITT). Compared with baseline, at one month post intervention, there were significant and clinically important differences between groups for MDP—Emotional Response, which favored the CPRP + SC group (Figure 3b, mean difference 5.9 ± 2.8 [95% CI 0.4 to 11.4], PP *p* = 0.05). At six months post intervention, significant and clinically important between-group differences were present for MDP—Immediate Perception (Figure 3a) (not significant with PP analysis *p* = 0.06), D-12 total score and D-12 physical subdomain score (Figure 3c,d) (remained significant with PP analysis *p* = 0.04 and *p* = 0.05 respectively). In each case, the CPRP + SC group improved breathlessness scores compared with CPRP + CBT where scores were relatively unchanged from baseline. Similarly, compared with baseline, there were a small number of within-group significant and clinically important differences, which consistently favored the CPRP + SC group. 

Respiratory related quality of life. Results of the ITT analysis of between- and within-group changes for CRQ domains are summarized in Table 2. Per-protocol analysis was consistent with ITT analysis (Supplementary Materials). Between groups, there were no statistically significant differences at follow-up assessments for CRQ subdomain scores. 

At one month post intervention, the mean difference between groups in CRQ-Dyspnea subdomain scores, while not statistically significant, exceeded the MID (0.6 ± 0.3 [−0.1 to 1.2]), reflecting small positive changes in the CPRP + CBT group compared with relatively larger negative changes in the CPRP + SC group. 

Habitual activity (accelerometry). Results of the ITT analysis of between- and within-group changes for accelerometry are summarized in Table 3. Per-protocol analysis was consistent with ITT analysis (Supplementary Materials). There were no statistically significant differences between groups at follow-up assessments for average minutes per day spent in sedentary, light or moderate/vigorous physical activity. Within-group differences generally presented an overall pattern for both groups where, compared with baseline, the average minutes per day spent sedentary increased across the 12-month follow-up period, while average time spent in light or MVPA decreased.

Self-reported time use (Multimedia Activity Recall for Adults and Children (MARCA)). Results of the ITT analysis of between- and within-group changes for MARCA are summarized in Table 4. Per-protocol analysis was consistent with ITT analysis (Supplementary Materials). Overall, there were very few differences in average daily time use for either group, with the exception of time spent in self care (CPRP + CBT relatively unchanged from baseline; CPRP + SC less time at 1- and 12-months post intervention and more time at 6 months post intervention).

Very few participants reported time spent in physical activity or work/study, resulting in a high percentage of zero values. Results of logistic regression (OR) conducted on their dichotomized values (0, ≥1) are reported in Table 4. Between-group differences were evident where, compared with baseline, the CPRP + CBT group were more likely to report time spent in physical activity (1- and 6-months post intervention) and less likely to report time spent in work/study (1 month post intervention).

### 3.3. Participant Feedback

Approximately half of the participants allocated to the CBT program provided written feedback on completion of the final CBT session (30/52, 57.6%). The majority of respondents supported the usefulness of the program, the group setting and the value of the group facilitator in attending the supervised exercise sessions (Supplementary Materials). Exit interviews were completed for 36 participants (CPRP + CBT *n* = 21; CPRP + SC *n* = 15) where 12 months after intervention completion, the majority of participants accurately identified their group allocation (CBT 76%; SC 80%). All interviewees unanimously supported the usefulness of pulmonary rehabilitation, satisfaction with study staff communication and participation in the study. Responses from CBT group participants were consistent with the feedback provided at the immediate end of the program. Responses from SC participants were more variable with respect to the enjoyment and value of the social group program (Supplementary Materials).

## 4. Discussion

In this very sedentary cohort of people living with COPD characterized by moderate-to-severe airflow obstruction, the addition to CPRP of a CBT intervention that targeted the sensation of breathlessness did not provide clear additional benefits for health outcomes beyond those achieved by adding a social group to standard CPRP at 1, 6 or 12 months post intervention. Between groups, there were no statistically significant or clinically important differences evident for the distance achieved in the 6MWT or anxiety/depression scores (primary outcomes). Sensations of breathlessness remained relatively unchanged at each time point for participants completing CPRP with the addition of CBT, whereas breathlessness sensations were improved at one (emotional response) and six months (physical sensations) for participants completing CPRP with social group control. There were few statistically significant or clinically important differences between groups for respiratory-related quality of life, time spent in and the nature of sedentary and physical activities, symptom severity scores and the number of hospital or emergency department presentations.

Given the breathlessness focus of the CBT intervention within this trial, the differences and direction of change between groups for breathlessness outcomes was unexpected. For instruments assessing ‘how breathlessness feels’ (MDP and D-12), a general pattern was evident across the three occasions of follow-up where, relative to baseline, the CPRP + SC group scores improved while the CPRP + CBT group remained relatively unchanged (excepting the MDP—emotional response at 12 months). This pattern underpinned the significant between-group differences for subdomain scores reflecting physical sensations of breathlessness six months after intervention (MDP—immediate perception, D-12 physical which is a large component of D-12 total). This reduction in the intensity of breathless sensations might be expected to be reflected in improved ratings for respiratory-related quality of life (CRQ-D) favoring the CPRP + SC. While mean within-group differences from baseline did not exceed the CRQ-D MID (0.5), CRQ-D mean scores improved in the CPRP + CBT group and deteriorated in the CPRP + SC group, underpinning the between-group differences at one month, which exceeded the MID (0.6 ± 0.3 [−0.1 to 1.2]. Whether the discrepancy between ‘how breathlessness feels’ and ‘how breathlessness impacts’ reflects a greater recognition and ‘tolerance’ of sensations within the CBT group because of frequent practice and reflection upon breathing sensations, or given the multiplicity of analyses, a random spurious finding, is unknown. 

In theory, effective psycho-educational interventions, such as CBT for breathless sensations or anxiety associated with exertional breathlessness, should reduce general anxiety, encourage greater participation in physical activity/exercise, and improve quality of life. In previous pooled analyses, when compared with usual care, there is evidence to support the positive effect of CBT interventions on health outcomes (anxiety, depression, breathlessness, exercise capacity and quality of life) [11]. However, future pooled analyses are likely to be impacted by the recent publication of the largest RCT comparing CBT (*n* = 242) with usual care (*n* = 181) in people living with COPD, which reported no significant differences between groups for HADs-A or D (and any secondary outcome) at six months post intervention (TANDEM RCT) [52]. 

Where CBT interventions are combined with and compared with pulmonary rehabilitation/exercise training for people living with COPD (controlled clinical trial [53]; RCTs [54,55,56,57,58]), very few significant between-group differences favoring the CBT intervention have been reported for: anxiety (Becks Anxiety Index (BAI) pre-post intervention) [56], depression (Becks Depression Index (BDI) pre-post intervention [56], time x treatment arm; HADs-D immediately post or three and six months post intervention) [55] or health-related quality of life (CRQ-Fatigue pre-post intervention [53]).

Where explanations have been proposed for the lack of a clear health advantage of adding a CBT intervention to pulmonary rehabilitation, these have included: (1) limited statistical power or insufficient sample sizes within analyses [53,54,55,56]; (2) absence of an attention/social group control [53,55]; (3) intensity/duration of the CBT intervention [54]; (4) dominant, health benefit of pulmonary rehabilitation (predictable improvement or slowing of deterioration [54,55]); (5) use of standardized versus personalized CBT approach [54]; (6) heterogeneity of psychological co-morbidities [54]; and (7) insufficient intervention duration/intensity to effect habitual behavioral changes [55].

Several alternative explanations exist. Firstly, as with all interventions, there are likely individuals for whom the intervention ‘works’ but within controlled trials, adding a CBT approach to pulmonary rehabilitation—irrespective of CBT focus or mode of delivery—does not provide a significant ‘optimized’ predictable group effect to the health outcomes achieved by pulmonary rehabilitation alone. Secondly, a reality raised by the authors of the TANDEM RCT [52] concerns the impact of comparatively brief CBT interventions being ‘too little, too late’ within the decades long, complex multimorbid disease trajectory of COPD. The participants in our trial reflected a cohort of very sedentary people with later stage disease, many of whom exhibited anxiety/depression with close to half of the trial cohort admitted to hospital for respiratory and non-respiratory related causes during the 12-month trial. Anxiety and depression, while highly prevalent among people with COPD, are unlikely to be solely attributable to chronic respiratory disease and will have contributing factors related to comorbid conditions and challenging socio-economic circumstances.

One further explanation concerns the high likelihood of the presence of ‘unlabeled’ or covert CBT within health professional-facilitated interactions (education and supervised breathlessness-inducing exercise sessions). Likely embedded in pulmonary rehabilitation are several CBT-based behavior change techniques (BCTs). For example, counselling directed toward changing health behaviors (e.g., uptake and creation of an exercise habit, encouragement of self-management, diet) is inherent in the interactions between staff and participants in pulmonary rehabilitation. These BCTs are often poorly or not described [59,60]. In this highly skilled workforce, it is likely that health professionals directly involved in day-to-day interactions with individuals use CBT principles in education, encouragement, and in responding to individual queries particularly related to breathlessness. For example, exercise and breathlessness-related anxiety, fear of exercise harm, clarification of beliefs, providing opportunities to ‘field test’ concerns and strategies. One of the practical difficulties in undertaking studies of psycho-cognitive interventions in complex interventions such as CPRP, especially in real-world settings, is the inability to completely quarantine the intervention, and the overlapping nature of therapeutic initiatives (specific communication approaches, BCTs, CBT principles) [61]. While there are empirically controlled trials exploring and supporting the benefits of specific communication approaches that are employed during exercise training (motivational interviewing, health coaching) [62] and evidence in phenomenological studies indicating the impact of health professional interaction on symptom and exercise beliefs [63], it is difficult to locate studies describing the nature of real-time individual therapist–participant interactions during education /exercise training sessions.

This trial was planned to be, and conducted within, an ecologically valid setting of a CPRP as delivered in the real world (pragmatic trial). This choice included referral processes, eligibility screening, use of standard assessments where possible (pre and post) and delivery of the CPRP. The additional assessments for this trial (breathlessness instruments, accelerometry and use-of-time interviews) were considerable. The standard assessments included the HADs to screen participants for anxiety and depression. While the HADs provides a generic assessment of anxiety and depression in people with somatic medical conditions and has been frequently used within previous studies of CBT interventions in people with COPD [11], it may not capture disease-specific aspects of anxiety in COPD. Recently, Christiansen et al. (2023) [64] have proposed a conceptual model of COPD-related anxiety derived from patient experiences. Factors included in this model, such as fear of dying, unsafe environments and fear-based avoidance behaviors are not reflected in HADs items, raising the possibility that HADs scores may underestimate the severity and prevalence of COPD-specific anxiety. Conducting the trial in a single center necessitated a randomization approach at the program (CPRP scheduled cycles) rather than the individual level, in order to eliminate crosstalk between participants allocated to different interventions arms. This block randomization approach, coupled with lower than anticipated study uptake in each CPRP cycle, extended the recruitment and data collection period. Consequently, when the ‘real-world’ health service introduced a symptom-limited cardiopulmonary exercise test as a basis for exercise prescription for exercise sessions with the CPRP, recruitment for this study ceased before reaching the target sample size.

While the findings of this study are generally consistent with more recent RCTs of pulmonary rehabilitation with and without the addition of CBT, there are several limitations. Missing data for study outcomes (especially for 6- and 12-month follow-up assessments, Supplementary Materials), fell short of the 31 persons per group estimated for sufficient power (mitigated by ITT). The CBT intervention was manualized and regular, informal debriefing conversations were held with the psychologist facilitating the CBT sessions, but rigorous fidelity assessments were not undertaken. In retrospect, the duration of the trial, and the number and type of assessment planned for participants was burdensome and likely to have contributed to a high attrition rate in both groups (CPRP + CBT = 38.5%; CPRP + SC= 42.8%). While most participants in both groups perceived that they had benefited from participation in CPRP, fidelity assessments of supervised exercise or education sessions were not undertaken and improvements in outcomes did not reach MIDs, though changes in 6MWD were comparable with similar Australian pragmatic center-based RCTs (mean change 10.82 [95% CI −4.52 to 26.16] [65], 14.7 [−5.7 to 35.1] [54]).

The trial described in this paper was in development for several years, leading up to the commencement of recruitment in 2011. At the time of planning this pragmatic RCT, foundational laboratory-based knowledge of the mechanism underlying breathless sensations was being progressively translated into clinical applications. Multidimensional instruments for breathlessness assessment were entering the clinical research arena. Breathlessness services were evolving [66,67], embedding evidence-based non-pharmacological strategies (i.e., handheld fan, cognitive approaches) and an assessment model founded on cognitive and behavioral reactions to breathlessness (Breathing Thinking Function model) [68,69]. Since this time, several standardized exercise tests for dyspnea have been developed [70] and the Breathing Thinking Function model for breathlessness has been included within the Australian Pulmonary Rehabilitation Toolkit (Lung Foundation Australia). While our study is likely underpowered for several health outcomes warranting a cautious acceptance of the findings, given the evolution of breathlessness services and inclusion of breathlessness management models within pulmonary rehabilitation landscapes, replicating this study is unlikely to provide additional clarity about the value of including CBT interventions with pulmonary rehabilitation.

## 5. Conclusions

Psychological approaches, such as CBT, can be an effective part of a management strategy for individuals living with chronic breathlessness. Whether the systematic addition of group-based CBT within complex interventions such as pulmonary rehabilitation provides benefit remains equivocal. In this single-center, pragmatic trial of people living with COPD, the addition of a CBT intervention targeting breathlessness to CPRP did not provide clear additional benefits for health outcomes beyond those achieved by adding a social group to standard CPRP at 1, 6, or 12 months post intervention. Although CBT principles and techniques are not typically listed as components of pulmonary rehabilitation, the degree to which they are present as active intervention components remains underexplored.

## Figures and Tables

**Table 1:** Participant characteristics at enrolment into the study. Data are mean ± standard deviation (SD) unless otherwise indicated.

			CPRP + CBT *n* = 52	CPRP + SC *n* = 49
Age (years)			71 ± 6	69 ± 10
Female: Male *n*=			28: 24	19: 30
Body mass index kg/m^2^			28 ± 7	27 ± 7
English spoken at home *n* (%)			52 (100)	46 (94)
Current smoker *n* (%)			10 (19)	6 (12)
Mini Mental State Examination			29.4 ± 1.3	29.0 ± 1.8
COTE-Index score			1.79 (2.55)	1.92 (2.77)
COTE score ≥ 4 *n* (%)			11 (21)	9 (18)
FEV_1_ percent predicted			48 ± 14	47 ± 19
FEV_1_/FVC			43 ± 13	42 ± 16
PaCO_2_ mmHg			40.5 ± 6.0	40.5 ± 6.0
PaO_2_ mmHg			73.8 ± 9.6	73.9 ± 11.7
GOLD Stage *n* (%)		2	25 (48)	22 (45)
		3	21 (40)	16 (33)
		4	6 (12)	11 (22)
Modified Medical Research Council dyspnea scale *n* (%) *		0	1 (2)	4 (8)
		1	27 (52)	15 (31)
		2	11 (21)	6 (12)
		3	11 (21)	12 (24)
		4	2 (4)	12 (24)
			*n* = 51	*n* = 46
Maximum distance 6MWD (m)			375 ± 127	374 ± 146
Before 6MWD	Perceived exertion		0.8 ± 1.0	1.0 ± 1.1
	MDP-A1 daily life		4.7 ± 2.1	4.8 ± 2.7
End 6MWD	Perceived exertion		3.4 ± 1.4	3.7 ± 2.0
	MDP-A1		4.3 ± 2.6	4.2 ± 2.9
			*n* = 49	*n* = 47
HADs-Anxiety		Score	7.1 ± 4.5	6.8 ± 4.5
		No case *n* (%)	28 (57)	27 (57)
		Borderline	10 (20)	7 (15)
		Case	11 (22)	13 (28)
HADs-Depression		Score	5.9 ± 4.0	6.6 ± 4.2
		No case *n* (%) *	33 (67)	27 (57)
		Borderline *	8 (16)	12 (26)
		Case *	1 (2)	8 (17)
Chronic Respiratory Questionnaire			*n* = 48	*n* = 46
		Dyspnea	4.5 ± 1.3	4.7 ± 1.5
		Fatigue	3.8 ± 1.2	3.9 ± 1.3
		Emotion	4.6 ± 1.2	4.8 ± 1.2
		Mastery	4.7 ± 1.3	4.9 ± 1.4
Habitual activity (accelerometry)			*n* = 43	*n* = 38
Mean minutes per day (awake time, excluding non-wear)		Sedentary	713.8 ± 111.6	726.2 ± 154.1
		Light	257.7 ± 94.4	244.3 ± 115.5
		MVPA#	7.1 ± 10.2	7.8 ± 9.5
Session attendance	Education sessions (max = 8)		6 ± 3	6 ± 3
	Exercise sessions (max = 16)		11 ± 5	11 ± 5
	CBT/social sessions (max = 8)		5 ± 3	5 ± 3
	Total sessions attended (max = 32)		21 ± 10	21 ± 9

COTE score = COPD-specific comorbidity test, CPRP = comprehensive pulmonary rehabilitation program, CBT = cognitive behavioral therapy, FEV_1_ % pred = forced expiratory volume in one second percent predicted, FEV1/FVC = ratio between FEV_1_ and forced vital capacity, 6MWD = 6 min walk distance, MDP-A1 = Multidimensional Dyspnea Profile-Affective Distress, HADs = Hospital Anxiety and Depression scale, MVPA = moderate-to-vigorous physical activity # time spent in vigorous activity per day < 1 min (*n* = 3 participants), SC—Social group control. * *p* ≤ 0.05.

**Table 2:** Respiratory-related quality of life outcomes for intention to treat analysis (ITT) within- and between-group differences from pre-intervention (baseline).

		Within-Group Differences from Baseline Mean (SE) [95% CI]		Between-Group Differences Mean (SE) [95% CI]
	Months Post Intervention	CPRP + CBT *n* = 52	CPRP + SC *n* = 49	CPRP + CBT vs. CPRP + SC [95% CI]
CRQ—Dyspnea MID 0.5 [43]	1	0.2 ± 0.2 [−0.3 to 0.6]	−0.4 ± 0.2 [−0.9 to 0.1]	0.6 ± 0.3 [−0.1 to 1.2]
	6	0.0 ± 0.2 [−0.4 to 0.5]	−0.3 ± 0.2 [−0.8 to 0.1]	0.3 ± 0.3 [−0.1 to 1.0]
	12	0.1 ± 0.2 [−0.3 to 0.5]	−0.1 ± 0.2 [−0.5 to 0.3]	0.2 ± 0.3 [−0.4 to 0.8]
CRQ—Emotion MID 0.5 [43]	1	0.1 ± 0.2 [−0.2 to 0.5]	0.3 ± 0.2 [0.0 to 0.7]	−0.2 ± 0.2 [−0.7 to 0.3]
	6	0.2 ± 0.2 [−0.1 to 0.6]	0.2 ± 0.2 [−0.2 to 0.6]	0.0 ± 0.2 [−0.5 to 0.5]
	12	0.0 ± 0.2 [−0.3 to 0.3]	−0.1 ± 0.2 [−0.5 to 0.2]	0.1 ± 0.2 [−0.3 to 0.6]
CRQ—Fatigue MID 0.5 [43]	1	0.1 ± 0.2 [−0.3 to 0.5]	0.2 ± 0.2 [−0.2 to 0.6]	−0.1 ± 0.3 [−0.7 to 0.4]
	6	0.1 ± 0.2 [−0.3 to 0.5]	−0.1 ± 0.2 [−0.5 to 0.3]	0.2 ± 0.3 [−0.4 to 0.8]
	12	0.2 ± 0.2 [−0.2 to 0.6]	−0.2 ± 0.0 [−0.6 to 0.2]	0.3 ± 0.3 [−0.2 to 0.9]
CRQ—Mastery MID 0.5 [43]	1	0.2 ± 0.2 [−0.2 to 0.6]	0.4 ± 0.2 [0.04 to 0.0]	−0.1 ± 0.3 [−0.7 to 0.4]
	6	0.3 ± 0.2 [0.0 to 0.7]	0.5 ± 0.2 [0.2 to 0.9] *p* = 0.01	−0.2 ± 0.3 [−0.7 to 0.3]
	12	0.2 ± 0.2 [−0.2 to 0.5]	−0.2 ± 0.2 [−0.5 to 0.2]	0.40 ± 0.3 [−0.1 to 0.9]

Data are mean, standard error (SE) and 95% confidence intervals adjusted for baseline values and covariates. CPRP—comprehensive pulmonary rehabilitation program, CBT—cognitive behavior therapy; CRQ—Chronic Respiratory Questionnaire, higher scores = better health related quality of life; MID = minimal important difference [43], SC—Social group control. Shaded cells indicate statistical difference *p* ≤ 0.05.

**Table 3:** Habitual activity (accelerometry) intention to treat analysis (ITT) within- and between group differences from pre-intervention (baseline).

		Within-Group Differences from Baseline Mean ± SE [95% CI]		Between-Group Differences Mean (SE) [95% CI]
Mean Minutes Per Day (Awake Time, Excluding Non-Wear)		CPRP + CBT *n* = 43	CPRP + SC *n* = 38	CPRP + CBT vs. CPRP + SC [95% CI]
Sedentary	Baseline	713.8 ± 111.6	726.2 ± 154.1	-
	1	39.3 ± 27.0 [−14.1 to 92.7]	−13.2 ± 30.0 [−72.7 to 46.3]	52.5 ±40.0 [−26.8 to 131.7]
	6	52.8 ± 28.1 [−2.8 to 108.4]	59.8 ± 30.5 [−0.6 to 120.2] *p* = 0.05	−7.0 ± 41.4 [−89.0 to 75.0]
	12	53.3 ± 27.7 [−1.6 to 108.1]	32.5 ± 28.9 [−24.8 to 89.8]	20.8 ± 40.2 [−58.8 to 100.3]
Light	Baseline	257.7 ± 94.4	244.3 ± 115.5	-
	1	−19.3 ± 11.5 [−42.1 to 3.5]	7.0 ± 12.9 [−18.5 to 32.5]	−26.3 ± 17.1 [−60.2 to 7.6]
	6	−26.9 ± 12.4 [−51.5 to −2.3] *p* = 0.03	−4.0 ± 13.4 [−30.6 to 22.6]	−22.9 ± 18.3 [−59.1 to 13.3]
	12	−50.5 ± 13.0 [−76.3 to −24.7] *p* = 0.0002	−35.1 ± 13.6 [−62.0 to −8.1] *p* = 0.01	−15.4 ± 18.9 [−52.9 to 22.0]
MVPA#	Baseline	7.1 ± 10.2	7.8 ± 9.5	-
	1	1.00 [0.78 to 1.29]	0.88 [0.68 to 1.12]	1.15 [0.81 to 1.62]
	6	0.68 [0.49 to 0.95] *p* = 0.02	0.82 [0.63 to 1.06]	0.83 [0.55 to 1.26]
	12	0.63 [0.45 to 0.88] *p* = 0.01	0.78 [0.60 to 1.01]	0.81 [0.53 to 1.23]

Data are mean, standard error (SE) and 95% confidence intervals adjusted for baseline values and other covariates except for MVPA# for which relative risks and 95% confidence intervals are presented. CPRP—comprehensive pulmonary rehabilitation program, CBT—cognitive behavior therapy, MVPA—moderate-to-vigorous physical activity (Poisson regression mixed model used for MVPA), SC—social group control. Shaded cells indicate statistical difference *p* ≤ 0.05.

**Table 4:** Multimedia Activity Recall for Adults and Children (MARCA) intention to treat analysis (ITT) within- and between-group differences from pre-intervention (baseline).

Superdomains		Within-Group Differences from Baseline Mean ± SE [95% CI]		Between-Group Differences Mean (SE) [95% CI]
Mean Minutes Per Day unless Otherwise Stated		CPRP + CBT *n* = 49	CPRP + SC *n* = 48	CPRP + CBT vs. CPRP + SC [95% CI]
Sleep	Baseline	493 ± 77	482 ± 74	-
	1	20.0 ± 12.8 [−5.3 to 45.3]	−11.8 ± 13.5 [−38.5 to 14.9]	31.8 ± 18.5 [−4.6 to 68.3]
	6	7.3 ± 13.3 [−18.9 to 33.5]	19.6 ± 13.5 [−7.1 to 46.2]	−12.2 ± 18.9 [−49.5 to 25.0]
	12	7.6 ± 13.7 [−19.4 to 34.6]	−5.5 ± 13.7 [−32.6 to 21.6]	13.1 ± 19.3 [−25.0 to 51.2]
Chores (indoor/outdoor)	Baseline	192 ± 97	173 ± 104	-
	1	−8.2 ± 14.5 [−36.9 to 20.5]	−9.2 ± 15.3 [−39.4 to 21.1]	0.9 ± 20.9 [−40.4 to 42.3]
	6	−13.6 ± 14.9 [−43.1 to 15.8]	−26.0 ± 15.2 [−56.0 to 4.0]	12.4 ± 21.2 [−29.5 to 54.3]
	12	−9.2 ± 14.8 [−38.4 to 20.0]	−34.3 ± 14.8 [−63.5 to −5.0] *p* = 0.02	25.1 ± 20.8 [−16.1 to 66.3]
Transport (passive, e.g., car)	Baseline	60 ± 37	52 ±35	-
	1	10.6 ± 6.7 [−2.6 to 23.8]	7.3 ± 7.1 [−6.6 to 21.3]	3.2 ±9.6 [−15.8 to 22.3]
	6	9.9 ± 6.8 [−3.6 to 23.4]	3.0 ± 7.0 [−10.7 to 16.8]	6.8 ± 9.7 [−12.4 to 26.1]
	12	1.0 ± 6.7 [−12.2 to 14.2]	3.8 ± 6.7 [−9.4 to 17.1]	−2.8 ± 9.4 [−21.5 to 15.8]
Screen time (television + computer use)	Baseline	218 ± 116	244 ± 109	
	1	−7.5 ± 15.1 [37.2 to 22.2]	23.6 ± 16.0 [−8.0 to 55.1]	−31.1 ± 21.8 [−74.0 to 11.9]
	6	15.9 ± 15.7 [−15.0 to 46.8]	−9.2 ± 15.9 [−40.7 to 22.3]	25.1 ± 22.3 [−18.9 to 69.1]
	12	1.9 ± 16.9 [−31.5 to 35.3]	34.9 ± 17.0 [1.4 to 68.5]	−33.0 ± 23.9 [−80.2 to 14.1]
Quiet time (reading /non reading)	Baseline	170 ± 86	158 ± 105	
	1	−25.4 ± 15.2 [−55.4 to 4.6]	−1.8 ± 16.1 [−33.6 to 30.0]	−23.6 ± 21.9 [−66.9 to 19.7]
	6	−10.9 ± 15.8 [−42.1 to 20.3]	19.6 ± 16.1 [−12.2 to 51.4]	−30.5 ± 22.5 [−74.9 to 13.9]
	12	0.2 ± 16.8 [−33.0 to 33.4]	12.2 ± 16.8 [−21.0 to 45.5]	−12.0 ± 23.7 [−58.8 to 34.8]
Self-care (grooming, bathing, eating)	Baseline	138 ± 27	149 ± 26	
	1	9.6 ± 6.4 [−2.9 to 22.1]	−12.7 ± 6.7 [−26.0 to 0.5]	22.3 ± 9.1 [4.3 to 40.4] *p* = 0.02
	6	10.1 ± 6.6 [−2.8 to 23.0]	18.0 ± 6.7 [−31.1 to −4.8] *p* = 0.01	28.1 ± 9.3 [9.7 to 46.5] *p* = 0.003
	12	0.4 ± 6.6 [−12.6 to 13.5]	−21.8 ± 6.6 [−34.9 to −8.8] *p* = 0.001	22.3 ± 9.3 [3.9 to 40.7] *p* = 0.02
Sociocultural (socializing, communicating, religious)	Baseline	104 ± 76	108 ± 47	
	1	1.3 ± 12.9 [−24.2 to 26.8]	−0.7 ± 13.6 [−27.6 to 26.2]	2.0 ± 18.6 [−34.7 to 38.7]
	6	−5.9 ± 13.4 [−32.2 to 20.5]	24.6 ± 13.6 [−2.3 to 51.5]	−30.4 ± 19.0 [−68.0 to 7.0]
	12	−2.0 ± 13.6 [−28.8 to 24.9]	4.9 ± 13.6 [−22.1 to 31.8]	−6.8 ± 19.2 [−44.7 to 31.1]
Physical activity (sports, exercise, active transport) #OR	Baseline	6 ± 15 (min/day)	9 ± 22 (min/day)	
	1	13.11 [4.40 to 39.11] *p* < 0.0001	1.13 [0.34 to 3.77]	11.59 [2.37 to 56.65] *p* = 0.003
	6	1.92 [0.70 to 5.25]	0.22 [0.06 to 0.80] *p* = 0.02	8.82 [1.71 to 45.52] *p* = 0.01
	12	2.55 [0.75 to 8.64]	3.04 [0.78 to 11.81]	0.84 [0.14 to 5.17]
Work/study (occupational, non-screen) #OR	Baseline	58 ± 80 (min/day)	65 ± 86 (min/day)	
	1	0.39 [0.14 to 1.11]	1.83 [0.60 to 5.53]	0.22 [0.05 to 0.97] *p* = 0.05
	6	1.00 [0.33 to 3.05]	0.72 [0.24 to 2.08]	1.39 [0.30 to 6.47]
	12	0.31 [0.11 to 0.93] *p* = 0.04	0.88 [0.30 to 2.57]	0.36 [0.08 to 1.61]

Data are mean, standard error (SE) and 95% confidence intervals adjusted for baseline values and other covariates. CPRP—comprehensive pulmonary rehabilitation program, CBT—cognitive behavior therapy, SC—social group control; #logistic regression, odds ratios (OR) where OR > 1.0 is more likely to accrue time, OR < 1 is less likely to accrue time. Shaded cells indicate statistical difference *p* ≤ 0.05.

## Data Availability

The data presented in this study are available on request from the corresponding author.

## Supplementary Materials

The following supporting information can be downloaded at: https://www.mdpi.com/article/10.3390/jcm12237286/s1, Table S1: Data availability per participant per assessment (raw data). Table S2: Health usage: Emergency department presentations and hospital admissions (12 months between date of pre-intervention assessment and final 12-month assessment). Table S3: Primary outcomes intention to treat analysis (ITT) fully adjusted models of between and within group differences from pre-intervention (baseline). Table S4: Primary outcomes per protocol (PP) analyses fully adjusted models of between and within group differences from pre-intervention (baseline). Table S5: Multidimensional breathlessness outcomes for intention to treat analysis (ITT) fully adjusted models within and between group differences from pre-intervention (Negative scores reflect improvement). Table S6: Multidimensional breathlessness outcomes for per protocol (PP) analysis of between and within group differences from pre-intervention (baseline). Table S7: Respiratory-related quality of life outcomes for intention to treat analysis (ITT) within and between-group differences from pre-intervention (baseline). Table S8: Respiratory related quality of life per protocol (PP) analysis of between and within group differences from pre-intervention (baseline). Table S9: Habitual activity (accelerometry) per protocol (PP) within and between group differences from pre-intervention (baseline). Table S10: Multimedia Activity Recall for Adults and Children (MARCA) super domains per protocol (PP) within and between group differences from pre-intervention (baseline). Table S11: Symptom diary (one-to-12-month post intervention assessment points). Table S12: Feedback from BREVE participants (*n* = 30) immediate end of intervention (anonymous written survey responses). Table S13: Exit interviews-conducted within one month post final 12 months assessment (by phone).
