# Supplementary material for: Pulmonary Rehabilitation with and without a Cognitive Behavioral Intervention for Breathlessness in People Living with Chronic Obstructive Pulmonary Disease: Randomized Controlled Trial
Source: J Clin Med. 2023 Nov 24;12(23):7286. doi: 10.3390/jcm12237286 (PMC10707579; doi:10.3390/jcm12237286)
Supplement: Supplementary file 1 [file jcm-12-07286-s001.zip › jcm-2708180-supplementary.pdf]

Supplementary Materials

Contents:

1. **Table S1:** Data availability per participant per assessment
2. **Table S2: Health usage:** Emergency department presentations and hospital admissions (12 months between date of pre-intervention assessment and final 12-month assessment).
3. Primary outcomes -Intention to treat (**Table S3**) and Per Protocol analysis (**Table S4**) fully adjusted models.
4. Multidimensional breathlessness assessments -Intention to treat (**Table S5**) and Per Protocol analysis (**Table S6**) fully adjusted models for Multidimensional Dyspnea Profile (MDP) and Dyspnoea-12(D-12).
5. Respiratory related quality of life - Intention to treat (**Table S7**) and Per Protocol analysis (**Table S8**) fully adjusted models for Chronic Disease Questionnaire (CRQ)
6. Habitual activity (accelerometery)- Per Protocol analysis (**Table S9**) fully adjusted models.
7. Multimedia Activity Recall for Adults and Children (MARCA) per protocol analysis (**Table S10**)
8. Symptom Diary (items and scoring and **Table S11)**
9. Feedback from BREVE participants (n=30) immediate end of intervention **(Table S12)**
10. Exit interviews **(Table S13)** -conducted within one month post final 12 months assessment (by phone)

Table S1: Data availability per participant per assessment (raw data)

	CPRP+CBT N=52 [%]				CPRP +SC N=49 [%]			
**Variable**	**Baseline N=52**	**1 month N=42**	**6 months N=36**	**12 months N=32**	**Baseline N=49**	**1 month N=36**	**6 months N=31**	**12 months N=28**
**HADs_A / HADs-D**	49 [94]	40 [77]	32 [62]	28 [54]	47 [96]	30 [61]	30 [61]	28 [57]
**6MWD**	51 [98]	34 [65]	27 [52]	25 [48]	46 [94]	31 [63]	24 [49]	22 [45]
**CRQ_Dyspnoea**	48 [92]	39 [75]	32 [62]	28 [54]	46 [94]	30 [61]	30 [61]	28 [57]
**CRQ_Fatigue**	48 [92]	39 [75]	31 [60]	28 [54]	46 [94]	30 [61]	30 [61]	28 [57]
**CRQ_Emotion**	48 [92]	39 [75]	31 [60]	28 [54]	46 [94]	30 [61]	30 [61]	28 [57]
**CRQ_Mastery**	48 [92]	39 [75]	31 [60]	28 [54]	46 [94]	30 [61]	30 [61]	28 [57]
**Multidimensional Dyspnea Profile**	52 [100]	40 [77]	33 [63]	29 [56]	49 [100]	34 [69]	30 [61]	28 [57]
**Dyspnoea-12**	52 [100]	40 [77]	33 [63]	29 [56]	49 [100]	34 [69]	30 [61]	28 [57]
**Accelerometry**	43 [83]	35 [67]	28 [54]	20 [38]	38 [78]	29 [59]	26 [53]	22 [45]
**MARCA**	49 [94]	37 [71]	32 [62]	30 [58]	48 [98]	34 [69]	30 [61]	28 [57]
**Symptom diary submitted n [%]**	25 [48]				19 [39]			

Key:

N=	Number of participants completed assessment	%	Percentage of participants originally randomised to group
CPRP	Comprehensive pulmonary rehabilitation program	CBT	Cognitive behaviour therapy
SC	Social group control	CRQ	Chronic Respiratory Questionnaire
HADS-A	Hospital Anxiety and Depression Score ANXIETY /DEPRESSION	HADS-D	Hospital Anxiety and Depression Score DEPRESSION
6MWD	Maximum distance achieved in six-minute walk test		
MARCA	Multimedia activity recall for children and adults (self-report)		

Table S2: Health usage: Emergency department presentations and hospital admissions (12 months between date of pre-intervention assessment and final 12-month assessment).

		CPRP+CBT N=52	CPRP +SC N=49	Between groups CPRP+ CBT versus CPRP +SC
**Emergency Department attendance #**	Number of participants	21 (40.4%)	25 (51.0%)	Chi^2 1.15, p= 0.28.
	Frequency of attendance mean (range)	2 (1 to 11)	3 (1 to 8)	
**Hospital admissions**- all causes #	Number of participants	20 (38.5%)	22 (44.8%)	
	Number of admissions mean (range)	3 (1 to 16)	3 (1 to 18)	Wilcoxon two sample test : P=0.88
	Total Days mean ± SD (range)	25 ± 47 (1 to 184)	19 ± 25 (1 to 91)	Wilcoxon two sample test : P=0.28
**Respiratory-related**	Number of participants	11	9	Number of participants admitted for different causes between groups not significantly different χ^2 (1, N = 64) =2.28, p=0.68).
	Frequency of admissions mean (range)	2 (1 to 5)	1 (1 to 2)	
	Days mean ± SD (range)	16 ± 14 (1 to 41)	9 ± 6 (4 to 24)	
**Cardiac-related**	Number of participants	6	7	
	Frequency of admissions mean (range)	1 (1 to 1)	2 (1 to 5)	
	Days mean ± SD (range)	2 ± 1 (1 to 4)	23 ± 28 (1 to 68)	
**Orthopaedic related**	Number of participants	1	0	
	Frequency of admissions mean (range)	4	-	
	Days mean ± SD (range)	-	-	
**Gastrointestinal-related**	Number of participants	5	3	
	Frequency of admissions mean (range)	1 (1 to 2)	1 (1 to 2)	
	Days mean ± SD (range)	21 ± 28 (1 to 67)	7 ± 10 (1 to 19)	
**Other**	Number of participants	8	14	
	Frequency of admissions mean (range)	3 (1 to 8)	3 (1 to 18)	
	Days mean ± SD (range)	24 ± 39 (1 to 113)	13 ± 23 (1 to 91)	

CPRP- Comprehensive pulmonary rehabilitation program, CBT - Cognitive behavior therapy, SC – Social group control.

Data (mean, standard deviation, range) reported for participants that had Emergency Department attendance or hospital admission rather than complete cohort.

Intention to treat and Per protocol analysis (fully adjusted models) for primary outcomes.

Table S3. Primary outcomes intention to treat analysis (ITT) fully adjusted models of between and within group differences from pre-intervention (baseline).

		**Within-group differences from baseline Mean ±SE [95% CI]**		**Between-group differences Mean (SE) [95% CI]**
Outcome	**Months post intervention**	CPRP + CBT N=52	CPRP + SC N=49	CPRP + CBT minus CPRP + SC [95% CI]
6MWD meters MID 30 m (95% CI 25 to 33) [35]	1	9.4 ± 9.5 [-9.5 to 28.2]	16.9 ± 9.9 [-2.7 to 36.4]	-7.5 ± 13.6 [-34.3 to 19.4]
	6	-7.3 ± 9.4 [-25.8 to 11.2]	4.2 ± 9.8 [-15.1 to 23.5]	-11.5 ± 13.5 [-38.1 to 15.1]
	12	-15.1 ± 8.2 [-31.4 to 1.1]	-11.3 ± 8.6 [-28.3 to 5.6]	-3.8 ± 11.9 [-27.2 to 19.6]
HADS –Anxiety MID -1.6 (-2.0 to -1.1) [37]	1	-0.5 ± 0.4 [-1.3 to -0.3]	-0.3 ± 0.4 [-1.1 to 0.6]	-0.3 ± 0.6 [-1.4 to 0.9]
	6	0.0 ± 0.4 [-0.8 to 0.8]	-1.1 ± 0.4 [-1.9 to -0.3] p=0.01	1.1 ± 0.6 [0.0 to 2.2]
	12	-0.7 ± 0.4 [-1.5 to 0.0)	-0.3 ± 0.4 [-1.0 to 0.5]	-0.4 ± 0.5 [-1.5 to 0.6]
HADS –Depression MID -1.6 (-1.8 to -1.5) [37]	1	-0.2 ± 0.4 [-1.0 to 0.5]	-0.4 ± 0.4 [-1.2 to 0.3]	0.2 ± 0.5 [-0.8 to 1.3]
	6	-0.1 ± 0.4 [-0.9 to 0.6]	-0.4 ± 0.4 [-1.1 to 0.4)	0.2 ± 0.5 [-0.9 to 1.3]
	12	0.0 ± 0.4 [-0.8 to 0.7]	0.6 ± 0.4 [-0.1 to 1.4]	-0.7 ± 0.5 [-1.7 to 0.4]

Data are mean, standard error (SE) and 95% confidence intervals, fully adjusted for baseline values and other covariates. CPRP- Comprehensive pulmonary rehabilitation program, CBT - Cognitive behavior therapy, SC – Social Group, HADs = Hospital Anxiety and Depression scale; MID = Minimum Important difference; 6MWD- Six-minute walk distance. Shaded cells indicate statistical difference p ≤ 0.05

Table S4. Primary outcomes per protocol (PP) analyses fully adjusted models of between and within group differences from pre-intervention (baseline).

		**Within-group differences from baseline Mean ±SE [95% CI]**		**Between-group differences Mean (SE) [95% CI]**
Outcome	**Months post intervention**	CPRP + CBT **N=44**	CPRP + SC **N=40**	CPRP + CBT minus CPRP + SC [95% CI]
6MWD meters MID 30 m (95%CI 25 to 33) [35]	1	9.2 ± 9.7 [-9.9 to 28.3]	16.9 ± 10.1 [-3.2 to 36.9]	-7.7 ± 13.9 [-35.1 to 19.7]
	6	-7.6 ± 9.5 [-26.3 to 11.2]	4.2 ± 10.0 [-15.5 to 23.9]	-11.8 ± 13.7 [-38.8 to 15.3]
	12	-15.3 ± 8.3 [-31.7 to 1.1]	-11.3 ± 8.7 [-28.5 to 5.9]	-4.0 ± 12.0 [-27.7 to 19.7]
HADS –Anxiety MID -1.6 (-2.0 to -1.1) [37]		**N=44**	**N=41**	
	1	-0.5 ± 0.4 [-1.4 to -0.3]	-0.2 ± 0.4 [-1.1 to 0.6]	-0.3 ± 0.6 [-1.5 to 0.9]
	6	0.0 ± 0.4 [-0.8 to 0.8]	-1.1 ± 0.4 [-1.9 to -0.3] p=0.01	1.0 ± 0.6 [-0.1 to 2.2]
	12	-0.7 ± 0.4 [-1.5 to 0.0]	-0.2 ± 0.4 [-1.0 to 0.5]	-0.5 ± 0.5 [-1.5 to 0.6]
HADS –Depression MID -1.6 (-1.8 to -1.5) [37]	1	-0.2 ± 0.4 [-1.0 to 0.5]	-0.5 ± 0.4 [-1.3 to 0.3]	0.3 ± 0.5 [-0.8 to 1.4]
	6	-0.2 ± 0.4 [-0.9 to 0.6]	-0.5 ± 0.4 [-1.3 to 0.3]	0.3 ± 0.6 [-0.8 to 1.4]
	12	0.0 ± 0.4 [-0.8 to 0.7]	0.5 ± 0.4 [-0.2 to 1.3]	-0.6 ± 0.5 [-1.6 to 0.5]

Data are mean, standard error (SE) and 95% confidence intervals, fully adjusted for baseline values and other covariates. CPRP- Comprehensive pulmonary rehabilitation program, CBT - Cognitive behavior therapy, SC – Social Group, HADs = Hospital Anxiety and Depression scale; MID = Minimum Important difference; 6MWD- Six-minute walk distance. Shaded cells indicate statistical difference p ≤ 0.05.

Table S5. Multidimensional breathlessness outcomes for intention to treat analysis (ITT) fully adjusted models within and between group differences from pre-intervention (Negative scores reflect improvement).

		Within-group differences from baseline Mean (SE) [95% CI]		Between-group differences Mean (SE) [95% CI]
**Breathlessness [Average over past two weeks]**	**Months post intervention**	CPRP + CBT N=52	CPRP + SC N=49	CPRP + CBT vs CPRP + SC [95% CI]
MDP-Affective Distress MID 0.82 (95% CI 0.56 to 1.08) [41]	1	0.0 ± 0.4 [-0.9 to 0.8]	-0.1 ± 0.5 [-1.0 to 0.8]	0.1 ± 0.6 [-1.2 to 1.3]
	6	0.0 ± 0.4 [-0.8 to 0.9]	-0.4 ± 0.5 [-1.3 to 0.5]	0.5 ± 0.6 [-0.8 to 1.7]
	12	-0.1 ± 0.4 [-1.0 to 0.8]	0.5 ± 0.4 [-0.4 to 1.4]	-0.6 ± 0.6 [-1.8 to 0.6]
MDP- Immediate Perception MID 4.63 (95% CI 3.21 to 6 .05) [41]	1	-0.5 ± 2.3 [-5.1 to 4.1]	-3.6 ± 2.5 [-8.5 to 1.3]	3.1 ±3.4 [-3.5 to 9.7]
	6	0.3 ± 2.3 [-4.3 to 4.9]	-6.4 ± 2.4 [-11.1 to -1.6] p=0.01	6.6 ±3.3 [0.0 to 13.2] p=0.05#
	12	1.6 ± 2.2 [-2.8 to 5.9]	-1.8 ± 2.2 [-6.2 to 2.6]	3.4 ± 3.1 [-2.8 to 9.6]
MDP- Emotional Response MID 2.37 (95%CI 1.10 to 3.64) [41]	1	2.3 ± 1.9 [-1.5 to 6.1]	-3.6 ± 2.0 [-7.6 to 0.4]	5.9 ±2.8 [0.4 to 11.4] p=0.04
	6	1.4 ± 1.9 [-2.3 to 5.2]	-3.7 ± 2.0 [-7.6 to 0.2]	5.1 ±2.7 [-0.3 to 10.4]
	12	3.6 ± 1.8 [0.1 to 7.0] p=0.05	0.6 ± 1.8 [-2.9 to 4.1]	3.0 ± 2.5 [-1.9 to 7.9]
D-12 Total MID 2.83 (95%CI 1.99 to 3.66) [41]	1	-0.5 ±1.5 [-3.3 to 2.4]	-2.9 ±1.6 [-6.0 to 0.2]	2.5 ± 2.1 [-1.7 to 6.7]
	6	0.1 ±1.5 [-2.8 to 2.9]	-4.3 ± 1.5 [-7.3 to -1.3] P=0.005	4.4 ± 2.1 [0.3 to 8.5] P=0.04
	12	1.4 ± 1.4 [-1.3 to 4.1]	-1.1 ± 1.4 [-3.8 to 1.6]	2.4 ± 1.9 [-1.4 to 6.2]
D-12 - Physical MID 1.81 (95%CI 1.29 to 2.34) [41]	1	-0.6 ± 0.9 [-2.4 to 1.2]	-1.9 ± 1.0 [-3.8 to 0.1]	1.3 ± 1.3 [-1.4 to 4.0]
	6	0.1 ± 0.9 [-1.7 to 1.9]	-2.6 ± 1.0 [-4.5 to -0.7] p=0.01	2.7 ± 1.3 [0.1 to 5.0] P=0.04
	12	0.6 ± 0.9 [-1.2 to 2.3]	-0.4 ± 0.9 [-2.2 to 1.3]	1.0 ± 1.2 [-1.5 to 3.4]
D-12 - Affective MID 1.07 (95%CI 0.64 to 1.49) [41]	1	-0.1 ± 0.7 [-1.2 to 1.5]	-1.1 ± 0.7 [-2.5 to 0.3]	1.2 ± 1.0 [-0.7 to 3.1]
	6	0.0 ±0.7 [-1.4 to 1.3]	-1.7 ± 0.7 [-3.1 to -0.3] p=0.01	1.7 ± 1.0 [-0.2 to 3.5]
	12	0.8 ± 0.6 [-0.4 to 2.0]	-0.7 ± 0.6 [-1.9 to 0.6]	1.5 ± 0.9 [-0.3 to 3.2]

Data are mean, standard error (SE) and 95% confidence intervals adjusted for baseline values. and other covariates. CPRP- Comprehensive pulmonary rehabilitation program, CBT -Cognitive behavior therapy; MDP- Multidimensional Dyspnea Profile; D-12 = Dyspnoea-12. For both MDP and D-12, higher rating/scores reflect worse/more intense sensation of breathlessness; MID= Minimal important difference as reported by Ekstrom et al 2020 [41] Shaded cells indicate statistical difference p ≤ 0.05 #Per protocol p=0.06

Table S6. Multidimensional breathlessness outcomes for per protocol (PP) analysis of between and within group differences from pre-intervention (baseline).

		Within-group differences from baseline Mean ±SE [95% CI]		Between-group differences Mean (SE) [95% CI]
**Secondary outcomes**	**Months post intervention**	CPRP + CBT **N=45**	CPRP + SC **N=43**	CPRP + CBT minus CPRP + SC [95% CI]
MDP-Affective Distress MID 0.82 (95% CI 0.56 to 1.08) [41]	1	-0.1 ± 0.4 [-0.9 to 0.8]	-0.1 ± 0.5 [-1.0 to 0.8]	0.1 ± 0.6 [-1.2 to 1.3]
	6	0.0 ± 0.4 [-0.8 to 0.9]	-0.4 ± 0.5 [-1.3 to 0.5]	0.5 ± 0.6 [-0.8 to 1.7]
	12	-0.1 ± 0.4 [-1.0 to 0.8]	0.5 ± 0.4 [-0.4 to 1.4]	-0.6 ± 0.6 [-1.8 to 0.6]
MDP-Immediate Perception MID 4.63 (95% CI 3.21 to 6 .05) [41]	1	-0.5 ± 2.4 [-5.2 to 4.1]	-3.5 ± 2.5 [-8.5 to 1.4]	3.0 ±3.4 [-3.7 to 9.8]
	6	0.3 ± 2.4 [-4.4 to 4.9]	-6.3 ± 2.5 [-11.2 to 1.4] p=0.01	6.6 ±3.4 [-0.1 to 13.3] P=0.06#
	12	1.6 ± 2.2 [-2.8 to 6.1]	-1.7 ± 2.3 [-6.2 to 2.8]	3.3 ± 3.2 [-3.0 to 9.6]
MDP- Emotional Response MID 2.37 (95%CI 1.10 to 3.64) [41]	1	2.3 ± 1.9 [-1.6 to 6.1]	-3.4 ± 2.1 [-7.5 to 0.8]	5.6 ±2.8 [0.0 to 11.2] P=0.05
	6	1.4 ± 1.9 [-2.4 to 5.2]	-3.4 ± 2.0 [-7.4 to 0.5]	4.8 ±2.8 [-0.6 to 10.3]
	12	3.6 ± 1.8 [0.0 to 7.1] p=0.05#	0.8 ± 1.8 [-2.8 to 4.3]	2.8 ± 2.5 [-2.2 to 7.8]
D-12 Total MID 2.83	1	-0.5 ± 1.5 [-3.4 to 2.5]	-2.9 ± 1.6 [-6.0 to 0.3]	2.4 ± 2.2 [-1.9 to 6.7]
	6	0.1 ± 1.5 [-2.8 to 3.0]	-4.2 ± 1.5 [-7.3 to -1.2]	4.3 ± 2.1 [0.1 to 8.5]

(95%CI 1.99 to 3.66) [41]			P=0.01	P=0.04
	12	1.4 ± 1.4 [-1.3 to 4.1]	-1.0 ± 1.4 [-3.8 to 1.7]	2.4 ± 1.9 [-1.4 to 6.2]
D-12 Physical MID 1.81 (95%CI 1.29 to 2.34) [41]	1	-0.6 ± 0.9 [-2.4 to 1.3]	-1.9 ± 1.0 [-3.9 to 0.1]	1.3 ± 1.4 [-1.4 to 4.0]
	6	0.1 ± 0.9 [-1.7 to 2.0]	-2.6 ± 1.0 [-4.5 to -0.7] P=0.01	2.7 ± 1.4 [0.0 to 5.4] P=0.05
	12	0.6 ± 0.9 [-1.2 to 2.3]	-0.4 ± 0.9 [-2.2 to 1.4]	1.0 ± 1.3 [-1.5 to 3.5]
D-12 Affective MID 1.07 (95%CI 0.64 to 1.49) [41]	1	0.1 ± 0.7 [-1.2 to 1.5]	-0.1 ± 0.7 [-2.5 to 0.4]	1.2 ± 1.0 [-0.8 to 3.1]
	6	-0.1 ± 0.7 [-1.4 to 1.3]	-1.6 ± 0.7 [-3.0 to -0.3] P=0.02	1.6 ± 1.0 [-0.3 to 3.5]
	12	0.8 ± 0.6 [-0.5 to 2.0]	-0.6 ± 0.6 [-1.9 to 0.6]	1.4 ± 0.9 [-0.4 to 3.2]

Data are mean, standard error (SE) and 95% confidence intervals adjusted for baseline values and other covariates. CPRP-Comprehensive pulmonary rehabilitation program, CBT -Cognitive behavior therapy; CRQ - Chronic Respiratory Questionnaire, higher scores = better health related quality of life; SC-Social Group; MDP- Multidimensional Dyspnea Profile; D-12 = Dyspnoea-12. For both MDP and D-12, higher rating/scores reflect worse/more intense sensation of breathlessness; MID= Minimal important difference as reported by Ekstrom et al 2020 [41]. Shaded cells indicate statistical difference p ≤ 0.05. #p≤0.05 intention to treat analysis.

Table S7. Respiratory-related quality of life outcomes for intention to treat analysis (ITT) within and between-group differences from pre-intervention (baseline)

		**Within-group differences from baseline Mean (SE) [95% CI]**		**Between-group differences Mean (SE) [95% CI]**
	**Months post intervention**	CPRP + CBT N=52	CPRP + SC N=49	CPRP + CBT vs CPRP + SC [95% CI]
CRQ – Dyspnoea MID 0.5 [43]	1	0.2 ± 0.2 [-0.3 to 0.6]	-0.4 ± 0.2 [-0.9 to 0.1]	0.6 ± 0.3 [-0.1 to 1.2]
	6	0.0 ± 0.2 [-0.4 to 0.5]	-0.3 ± 0.2 [-0.8 to 0.1]	0.3 ± 0.3 [-0.1 to 1.0]
	12	0.1 ± 0.2 [-0.3 to 0.5]	-0.1 ± 0.2 [-0.5 to 0.3]	0.2 ± 0.3 [-0.4 to 0.8]
CRQ- Emotion MID 0.5 [43]	1	0.1 ± 0.2 [-0.2 to 0.5]	0.3 ± 0.2 [0.0 to 0.7]	-0.2 ± 0.2 [-0.7 to 0.3]
	6	0.2 ± 0.2 [-0.1 to 0.6]	0.2 ± 0.2 [-0.2 to 0.6]	0.0 ± 0.2 [-0.5 to 0.5]
	12	0.0 ± 0.2 [-0.3 to 0.3]	-0.1 ± 0.2 [-0.5 to 0.2]	0.1 ± 0.2 [-0.3 to 0.6]
CRQ- Fatigue MID 0.5 [43]	1	0.1 ± 0.2 [-0.3 to 0.5]	0.2 ± 0.2 [-0.2 to 0.6]	-0.1 ± 0.3 [-0.7 to 0.4]
	6	0.1 ± 0.2 [-0.3 to 0.5]	-0.1 ± 0.2 [-0.5 to 0.3]	0.2 ± 0.3 [-0.4 to 0.8]
	12	0.2 ± 0.2 [-0.2 to 0.6]	-0.2 ± 0.0 [-0.6 to 0.2]	0.3 ± 0.3 [-0.2 to 0.9]
CRQ- Mastery MID 0.5 [43]	1	0.2 ± 0.2 [-0.2 to 0.6]	0.4 ± 0.2 [0.04 to 0.0]	-0.1 ± 0.3 [-0.7 to 0.4]
	6	0.3 ± 0.2 [0.0 to 0.7]	0.5 ± 0.2 [0.2 to 0.9] p=0.01	-0.2 ± 0.3 [-0.7 to 0.3]
	12	0.2 ± 0.2 [-0.2 to 0.5]	-0.2 ± 0.2 [-0.5 to 0.2]	0.40 ± 0.3 [-0.1 to 0.9]

Data are mean, standard error (SE) and 95% confidence intervals adjusted for baseline values and other covariates. CPRP- Comprehensive pulmonary rehabilitation program, CBT -Cognitive behavior therapy; CRQ - Chronic Respiratory Questionnaire, higher scores = better health related quality of life; MID= Minimal important difference Schünemann et al 2020 [43]; SC-Social group. Shaded cells indicate statistical difference p ≤ 0.05.

Table S8. Respiratory related quality of life per protocol (PP) analysis of between and within group differences from pre-intervention (baseline).

		**Within-group differences from baseline Mean ±SE [95% CI]**		**Between-group differences Mean (SE) [95% CI]**
**Secondary outcomes**	**Months post intervention**	CPRP + CBT **N=43**	CPRP + SC **N=40**	CPRP + CBT minus CPRP + SC [95% CI]
CRQ-Dypsnoea MID 0.5 [43]	1	0.2 ± 0.2 [-0.3 to 0.6]	-0.4 ± 0.2 [-0.9 to 0.1]	0.6 ± 0.3 [-0.1 to 1.2]
	6	0.0 ± 0.2 [-0.4 to 0.5]	-0.3 ± 0.2 [-0.8 to 0.1]	0.4 ± 0.3 [-0.3 to 1.0]
	12	0.1 ± 0.2 [-0.3 to 0.5]	-0.1 ± 0.2 [-0.5 to 0.3]	0.2 ± 0.3 [-0.4 to 0.8]
CRQ-Emotion MID 0.5 [43]	1	0.1 ± 0.2 [-0.2 to 0.5]	0.3 ± 0.2 [0.0 to 0.7]	-0.2 ± 0.3 [-0.7 to 0.3]
	6	0.2 ± 0.2 [-0.1 to 0.6]	0.2 ± 0.2 [-0.2 to 0.6]	0.0 ± 0.3 [-0.5 to 0.5]
	12	0.0 ± 0.2 [-0.3 to 0.4]	-0.1 ± 0.2 [-0.5 to 0.2]	0.1 ± 0.2 [-0.3 to 0.6]
CRQ- Fatigue MID 0.5 [43]	1	0.1 ± 0.2 [-0.3 to 0.5]	0.2 ± 0.2 [-0.2 to 0.6]	-0.2 ± 0.3 [-0.7 to 0.4]
	6	0.1 ± 0.2 [-0.3 to 0.5]	-0.1 ± 0.2 [-0.5 to 0.3]	0.2 ± 0.3 [-0.4 to 0.8]
	12	0.2 ± 0.2 [-0.2 to 0.6]	-0.2 ± 0.2 [-0.6 to 0.2]	0.3 ± 0.3 [-0.2 to 0.9]
CRQ- Mastery MID 0.5 [43]	1	0.2 ± 0.2 [-0.2 to 0.6]	0.3 ± 0.2 [-0.1 to 0.7]	-0.1 ± 0.3 [-0.7 to 0.4]
	6	0.3 ± 0.2 [-0.1 to 0.7]	0.5 ± 0.2 [0.1 to 0.9] P=0.01#	-0.2 ± 0.3 [-0.7 to 0.3]
	12	0.2 ± 0.2 [-0.2 to 0.5]	-0.2 ± 0.2 [-0.5 to 0.2]	0.4 ± 0.3 [-0.1 to 0.9]

Data are mean, standard error (SE) and 95% confidence intervals adjusted for baseline values and other covariates. CPRP- Comprehensive pulmonary rehabilitation program, CBT -Cognitive behavior therapy; CRQ - Chronic Respiratory Questionnaire, higher scores = better health related quality of life; MID= Minimal important difference Schünemann et al 2020 [43]; SC-Social group. Shaded cells indicate statistical difference p ≤ 0.05.

Table S9. Habitual activity (accelerometry) per protocol (PP) within and between group differences from pre-intervention (baseline).

		**Within-group differences from baseline Mean ±SE [95% CI]**		**Between-group differences Mean (SE) [95% CI]**
Mean minutes per day (awake time, excluding non-wear)		CPRP + CBT **N=39**	CPRP **N= 34**	CPRP + CBT vs CPRP [95% CI]
Sedentary	Baseline	713.8 ± 111.6	726.2 ± 154.1	-
	1	38.7 ± 27.4 [-15.6 to 93.0]	-16.7 ± 30.9 [-77.9 to 44.5]	55.4 ± 41.0 [-25.8 to 136.5]
	6	52.3 ± 28.4 [-4.0 to 108.7]	56.4 ± 31.3 [-5.7 to 118.4]	-4.1 ± 42.3 [-87.8 to 79.7]
	12	52.5 to 28.0 [-3.1 to 108.0]	29.9 ± 29.5 [-28.6 to 88.4]	22.6 ± 40.8 [-58.3 to 103.4]
Light	Baseline	257.7 ± 94.4	244.3 ± 115.5	-
	1	-19.0 ± 11.7 [-42.1 to 4.1]	7.9 ± 13.2 [-18.3 to 34.0]	-26.9 ± 17.5 [-61.5 to 7.7]
	6	-26.6 ± 12.6 [-51.5 to -1.8] p=0.04	-3.2 ± 13.8 [-30.5 to 24.1]	-23.5 ± 18.6 [-60.4 to 13.4]
	12	-50.1 ± 13.2 [-76.3 to -23.9] P =0.0002	-34.3 ± 14.0 [-61.9 to -6.6] P=0.02	-15.8 ± 19.3 [-54.0 to 22.3]
MVPA#	Baseline	7.1 ± 10.2 (min/day)	7.8 ± 9.5 (min/day)	-
	1	1.01 [0.78 to 1.29]	0.89 [0.69 to 1.14]	1.13 [0.80 to 1.60]
	6	0.68 [0.49 to 0.95] P=0.03	0.84 [0.65 to 1.09]	0.81 [0.53 to 1.24]
	12	0.64 [0.45 to 0.89] P=0.01	0.80 [0.61 to 1.04]	0.80 [0.52 to 1.23]

Data are mean, standard error (SE) and 95% confidence intervals adjusted for baseline values and other covariates except for MVPA where estimates are Relative Risk (95% CI) obtained from Poisson regression models. CPRP- Comprehensive pulmonary rehabilitation program, CBT -Cognitive behavior therapy; SC- Social group. MVPA - Moderate to vigorous physical activity (#Poisson models with estimates reported as relative risk (95% CI).

Table S10. Multimedia Activity Recall for Adults and Children (MARCA) super domains per protocol (PP) within and between group differences from pre-intervention (baseline).

Superdomains		Within-group differences from baseline Mean ±SE [95% CI]		Between-group differences Mean (SE) [95% CI]
Mean minutes per day		CPRP + CBT **N=49**	CPRP + SC **N= 48**	CPRP + CBT vs CPRP + SC [95% CI]
Sleep	Baseline	493 ± 77	482 ± 74	-
	1	19.7 ± 12.8 [-5.6 to 45.0]	-15.6 ± 13.7 [-42.7 to 11.4]	35.4 ± 18.6 [-1.4 to 72.1]
	6	6.8 ± 13.3 [-19.5 to 33.0]	15.5 ± 13.7 [-11.5 to 42.5]	-8.7 ± 19.0 [-46.3 to 28.8]
	12	7.3 ± 13.8 [-19.9 to 34.5]	-9.9 ± 14.0 [-37.5 to 17.8]	17.2 ± 19.6 [-21.5 to 55.8]
Chores (indoor/outdoor)	Baseline	192 ± 97	173 ± 104	-
	1	-8.2 ± 14.7 [-37.2 to 20.8]	-8.7 ± 15.7 [-39.7 to 21.2]	0.5 ± 21.3 [-41.6 to 42.7]
	6	-13.8 ± 15.1 [-43.5 to 16.0]	-25.7 ± 15.6 [-56.5 to 5.0]	12.0 ± 21.6 [-30.7 to 54.7]
	12	-9.3 ± 14.9 [-38.8 to 20.1]	-34.1 ± 15.1 [-63.9 to -4.2] p=0.03	24.7 ± 21.2 [-17.1 to 66.6]
Transport (Passive, e.g. car)	Baseline	60 ± 37	52 ±35	-
	1	10.7 ± 6.8 [-2.6 to 24.1]	8.1 ± 7.2 [-6.2 to 22.3]	2.7 ± 9.8 [-16.7 to 22.0]
	6	9.9 ± 6.9 [-3.7 to 23.6]	3.8 ± 7.1 [-10.2 to 17.9]	6.1 ± 9.9 [-13.4 to 25.6]
	12	1.1 ± 6.7 [-12.2 to 14.4]	4.6 ± 6.8 [-8.9 to 18.1]	-3.5 ± 9.6 [-22.4 to 15.4]
Screen time (Television + Computer use)	Baseline	218 ± 116	244 ± 109	
	1	-7.4 ± 15.2 [37.4 to 22.6]	23.1 ± 16.3 [-8.9 to 55.2]	-30.5 ± 22.1 [-74.1 to 13.1]
	6	16.0 ± 15.8 [-15.2 to 47.2]	-9.6 ± 16.2 [-41.7 to 22.4]	25.7 ± 22.6 [-19.0 to 70.3]
	12	2.0 ± 17.1 [-31.7 to 35.8]	34.4 ± 17.4 [0.1 to 68.6]	-32.3 ± 24.3 [-80.3 to 15.7]
Quiet time (Reading /non reading)	Baseline	170 ± 86	158 ± 105	
	1	-25.4 ± 15.3 [-55.7 to 4.9]	-1.0 ± 16.4 [-33.4 to 31.4]	-24.5 ± 22.3 [-68.5 to 19.6]
	6	-10.8 ± 15.9 [-42.3 to 20.7]	20.4 ± 16.4 [-12.0 to 52.8]	-31.2 ± 22.8 [-76.2 to 13.9]
	12	0.2 ± 17.0 [-33.3 to 33.8]	13.2 ± 17.3 [-20.9 to 47.3]	-12.9 ± 24.1 [-60.6 to 34.7]
Self-care (Grooming, bath-ing, eating)	Baseline	138 ± 27	149 ± 26	
	1	9.6 ± 6.4 [-3.1 to 22.3]	-13.1 ± 6.9 [-26.6 to 0.5]	22.7 ± 9.3 [4.2 to 41.1] P=0.02
	6	10.1 ± 6.6 [-3.0 to 23.2]	18.3 ± 6.8 [-31.8 to -4.8] P=0.01	28.4 ± 9.5 [9.6 to 47.2] P=0.003
	12	0.4 ± 6.7 [-12.8 to 13.6]	-22.2 ± 6.8 [-35.6 to -8.8] P=0.001	22.6 ± 9.5 [3.8 to 41.3] P=0.02
Sociocultural (Socializing, communicating, religious)	Baseline	104 ± 76	108 ± 47	
	1	1.8 ± 13.0 [-23.9 to 27.5]	-0.4 ± 13.9 [-27.8 to 27.1]	2.1 ± 18.9 [-35.2 to 39.5]
	6	-5.4 ± 13.5 [-32.0 to 21.3]	25.0 ± 13.9 [-2.4 to 52.4]	-30.4 ± 19.3 [-68.5 to 7.8]
	12	-1.5 ± 13.8 [-28.7 to 25.6]	5.3 ± 14.0 [-22.3 to 32.9]	-6.8 ± 19.5 [-45.4 to 31.8]
Physical Activity (Sports, exercise, active transport) #OR	Baseline	6 ± 15 (min/day)	9 ±22 (min/day)	
	1	2.55 ± 0.56 [1.45 to 3.65] P < 0.0001	0.11 ±0.62 [-1.11 to 1.32]	11.50 [2.32 to 57.03] P=0.003
	6	0.64 ± 0.52 [-0.38 to 1.66]	-1.52 ± 0.67 [-2.84 to -0.21] P=0.02	8.71 [1.66 to 45.55] P=0.01
	12	0.92 ± 0.62 [-0.31 to 2.16]	1.09 ± 0.69 [-0.28 to 2.46]	0.85 [0.13 to 5.33]
Work/Study (Occupational, non-screen) #OR	Baseline	58 ±80 (min/day)	65 ±86 (min/day)	
	1	0.38 [0.14 to 1.09]	1.92 [0.63 to 5.86]	0.20 [0.04 to 0.91] P=0.04
	6	0.97 [0.31 to 2.98]	0.76 [0.26 to 2.20]	1.28 [0.27 to 6.00]
	12	0.31 [0.10 to 0.92] P=0.03	0.94 [0.32 to 2.74]	0.33 [0.07 to 1.49]

Data are mean, standard error (SE) and 95% confidence intervals adjusted for baseline values and other covariates. CPRP-Comprehensive pulmonary rehabilitation program, CBT -Cognitive behavior therapy, SC-Social group, #Logistic regression, Odds ratios (OR) where OR >1.0 more likely to accrue time, OR <1 less likely to accrue time.

Symptom Diary (items and scoring guidelines).

Symptom diaries format and scoring based on Effing *et al. 2009 [Ref 46.]*

Symptom diaries (month to a page) were provided to participants at the one-month post intervention assessment. Monthly phone calls to each participant were scheduled to encourage diary completion. Symptoms diaries could be submitted at the end of each month (month page) or sequentially at the six and 12 months follow up assessments.

For each day or each month, participants were requested to indicate whether their usual symptoms had changed (Section A). If NO, no further responses were required. If YES, participants indicated which symptoms and the nature of the change [if any, Section B) and whether health care was sought, or antibiotics/prednisolone commenced (Section C). Scores for each section are presented below. Data compiled from symtom diaries for each participant included:

- Total number of calendar days completed.
- Total number of days where symptoms were unchanged.
- Total number of days symptoms changed.
- Symptom severity score per day (summed score per day, range 0 to 11)
- Average Symptom Severity score (total number of calendar days completed]
- Average maximum Symptom Severity score (total number of calendar days completed]
- Hospital admission days (scored 15 per day of admission).

	Questions	Response options	Score
Section A	Did you have more symptoms than usual during the last 24 hours?	YES	**1**
		No	0
Section B	Sputum production	No more than usual	0
		Slightly more than usual	1
		Significantly more than usual	**2**
	Sputum colour	Usual for me	0
		Different from usual	**2**
	Breathlessness	No more than usual	0
		Slightly more than usual	1
		Significantly more than usual	**2**
	Did you have a fever (>38.5C) or did you experience a significant change in coughing and /or wheezing in the last 24 hours?	No	0
		Yes	**1**
Section C	If your symptoms changed did you…		
	Visit your GP?	Yes	1
		No	0
	Visit an emergency department?	Yes	1
		No	0
	Get admitted to hospital?	Yes	**15**
		No	Score each day of admission 15 irrespective of other items.
	Start a course of antibiotics or prednisone?	Yes	1
		No	0

Table S11: Symptom diary (one-to-12-month post intervention assessment points)
Participants were provided with a 12-month diary (month per page) at the one -month post intervention assessment and invited to complete the diary daily until the final 12-month post intervention assessment (~8 months). A small number of participants contributed data for 12 rather than eight months.

	**CPRP+CBT N=52 [%]**	**CPRP +SC N=49 [%]**
**Submitted n [%]**	25 [48]	19 [39]
**Days with recorded data** Mean SD [range]	249 ±132 [13 to 366]	320 ± 73 [99 to 365]
**Days where symptoms did not change** (% out of days with recorded data)	77 ± 29%	78 ±16%
**Symptom severity score** (average of days with recorded data) No significant difference between groups, p = 0.81	1.80 ± 1.93 [1.00 to 2.60]	1.93 ± 1.30 [1.30 to 2.56]

Table S12: Feedback from BREVE participants (n=30) immediate end of intervention (anonymous written survey responses)

Questionnaire items	Strongly agree	Agree	Disagree	Strongly disagree	Unable to recall /give response	Strongly agree/ Agree. [%]	Strongly disagree /Disagree. [%]	Unable to recall /give response [%]
1: I found the BREVE program useful	19	10			1	96.7	0.0	3.3
2: I feel I have a better understanding of my sensation of breathlessness	18	11			1	96.7	0.0	3.3
3: I feel I can manage my sensation of breathlessness better	16	12	1		1	93.3	3.3	3.3
4: I feel I can exercise for longer without my sensation of breathlessness troubling me	11	14	2		3	83.3	6.7	10.0
5: I am less anxious about my sensation of breathlessness when I exercise	16	12	1		1	93.3	3.3	3.3
6: Thinking about the thoughts I have when I am breathless has helped me	9	17	2		2	86.7	6.7	6.7
7: There was too many homework tasks in the BREVE program	3	6	13	7	1	30.0	66.7	3.3
8: The information provided in the BREVE program was too complex	1	2	16	9	2	10.0	83.3	6.7
9: The BREVE coach helped me understand the material in the BREVE book	19	10			1	96.7	0.0	3.3
10: I enjoyed the BREVE group sessions	22	6			2	93.3	0.0	6.7
11: I appreciated having the BREVE coach work with me during the supervised exercise sessions	25	5				100.0	0.0	0.0
12: The BREVE program does not need a group session as I could have worked through the BREVE book on my own		2	13	14	1	6.7	90.0	3.3

Table S13: Exit interviews -conducted within one month post final 12 months assessment (by phone)

Number of individuals attending 12 months post intervention assessment [as allocated]	**CPRP +CBT N=32**	**CPRP N=28**
Number of individuals interviewed [%] Females :Males	21 [65.6%] 15:6	15 [53.6%] 6:9
Were you in the group that met on Friday and talked about the way you thought about the sensation of breathlessness or the group that met on Friday and talked about a variety of things?		
Identified as allocated (accurate)	16	12
Identified as being in alternate group (inaccurate)	2	1
Unable to recall /not able to provide a response	3	2

	CPRP + CBT n=21					CPRP + SC n=15					CPRP + CBT %			CPRP + SC %		
	SA	A	D	SD	UtR	SA	A	D	SD	UtR	SA/A	SD/D	UtR	SA/A	SD/D	UtR
The pulmonary rehabilitation lectures and exercise classes were useful	12	9				10	5				100	0	0	100	0	0
I found the BREVE /social group program useful	6	15				1	10	4			100	0	0	73	27	0
I feel I have a better understanding of my sensation of breathlessness	9	12				6	8	1			100	0	0	93	7	0
I feel I can manage my sensation of breathlessness better	7	12			2	7	6	2			90	0	10	87	13	0
I feel I can exercise for longer without my sensation of breathlessness troubling me	4	12	5			2	6	7			76	24	0	53	47	0
I am less anxious about my sensation of breathlessness when I exercise	5	9	4		3	4	6	4	1		67	19	14	67	33	0
I enjoyed the BREVE /social group program	7	13			1						95	0	5	73	20	7
**BREVE only -** Thinking about the thoughts I have when I am breathless has helped me	4	11	1		5						71	5	24			
**BREVE only** - There were too many homework tasks in the BREVE program	1	7	13								38	62	0			
**BREVE only -** The information provided in the BREVE program was too complex		2	18	1							10	90	0			
**BREVE only** - The BREVE coach helped me understand the material in the BREVE book	6	15									100	0	0			
**BREVE only -** I appreciated have the BREVE coach work with me during the supervised exercise sessions	4	16			1						95	0	5			
**BREVE only -** The BREVE program does NOT need a group session as I could have worked through the BREVE book on my own	1	1	15	3	1						10	86	5			
**SOCIAL group only**- Meeting people in a social setting was enjoyable						3	11	1						93	7	0
**SOCIAL group only** - Talking to people in the social group helped me change the way I see myself						1	6	8						47	53	0
**Symptom diary -** Recording information every day was difficult	1	7	9		4	1	4	9		1	38	43	19	33	60	7
**Symptom diary -**This diary was too complex to understand	1	2	14	1	3	1	13			1	14	71	14	93	0	7
**Symptom diary** - Recording information every day gave me a useful record	2	12	3	1	3		12	2		1	67	19	14	80	13	7
**Individualized report** -The report sent to me about some of the assessment over the past 12 months was useful	3	15	1		2	1	8	2		4	86	5	10	60	13	27
Overall, I was satisfied with **communication between the study staff and myself**	9	12				6	9				100	0	0	100	0	0
Overall, I was glad I participated in this study	8	13				9	6				100	0	0	100	0	0
Overall, I think participating in the pulmonary rehabilitation and BREVE /Social group has helped me	11	10				7	6	1		1	100	0	0	87	7	7

SA= Strongly agree, **A**=Agree, **D**=Disagree, **SD**= Strongly disagree, **UtR** = Unable to recall /respond
